# Fundamental aspects of symmetry and order parameter coupling for martensitic transition sequences in Heusler alloys

**DOI:** 10.1107/S2052520618012970

**Published:** 2018-11-14

**Authors:** Michael A. Carpenter, Christopher J. Howard

**Affiliations:** a Department of Earth Sciences, University of Cambridge, Downing Street, Cambridge, CB2 3EQ, UK; b School of Engineering, University of Newcastle, Callaghan, NSW 2308, Australia

**Keywords:** martensite, phase transitions, group theory, Heusler alloys, order parameters

## Abstract

Martensitic phase transitions in which there is a group–subgroup relationship between the parent and product structures are driven by combinations of soft-mode and electronic instabilities. These have been analysed from the perspective of symmetry, by considering possible order parameters operating with respect to a parent structure which has space group . Heusler structures with different stoichiometries are derived by operation of order parameters belonging to irreducible representations and P1 to describe the atomic ordering configurations. Electronic instabilities are ascribed to an order parameter belonging to the Brillouin zone centre, , which couples with shear strains to give tetragonal and orthorhombic distortions. An additional zone centre order parameter, with  symmetry, is typically a secondary order parameter but in some cases may drive a transition. Soft-mode instabilities produce commensurate and incommensurate structures for which the order parameters have symmetry properties relating to points along the Σ line of the Brillouin zone for the cubic *I* lattice. The electronic and soft-mode order parameters have multiple components and are coupled in a linear–quadratic manner as . As well as providing comprehensive tables setting out the most important group–subgroup relationships and the order parameters which are responsible for them, examples of NiTi, RuNb, Ti_50_Ni_50−*x*_Fe*_x_*, Ni_2+*x*_Mn_1−*x*_Ga and Ti_50_Pd_50−*x*_Cr*_x_* are used to illustrate practical relevance of the overall approach. Variations of the elastic constants of these materials can be used to determine which of the multiple order parameters is primarily responsible for the phase transitions that they undergo.

## Abstract 2

The combinations of phase transitions which occur in Heusler alloys in terms of order parameters and symmetry have been analysed using a group theoretical approach. It is shown how this approach can be applied to relevant examples.

## 1. Introduction   

Ferroelastic phase transitions in functional oxides are accompanied by symmetry-breaking shear strains which typically fall in the range ∼0.1–5% (Salje, 1993 ▸; Carpenter *et al.*, 1998 ▸). Most can be understood in terms of some structural or electronic instability with a driving order parameter that gives rise to the strain by coupling. Although the strength of coupling between individual strain components, *e_i_*, and the order parameter, *Q*, is a material property, its form, λ*e_i_Q*, *λe_i_Q* ^2^, λ*e_i_* ^2^ *Q*, λ*e_i_* ^2^ *Q* ^2^ …, depends on symmetry and is determined by rigorous group theoretical rules. The same symmetry rules apply to coupling between two or more order parameters in materials with multiple instabilities, and the form of this coupling determines how, for example, multiferroic materials may respond to an external electric or magnetic field. As set out for the cases of transitions in perovskites driven by combinations of octahedral tilting, ferroelectric displacements, atomic ordering and cooperative Jahn–Teller distortions, the group theory program *ISOTROPY* (Stokes *et al.*, 2007 ▸) has allowed such relationships to be tabulated even for the most complex cases (Howard & Stokes, 1998 ▸, 2004 ▸, 2005 ▸; Stokes *et al.*, 2002 ▸; Carpenter & Howard, 2009 ▸).

Martensitic transitions in which there is a group/subgroup relationship between parent and product structures, such as in the cases of Heusler compounds and shape memory alloys based on NiTi, may appear to be different because of the much larger shear strains involved (typically ≥ 10%), but they are still essentially ferroelastic. Multiple instabilities are also characteristic and the relevant order parameters relate to atomic ordering, band Jahn–Teller effects, magnetic ordering, superconductivity and soft modes. This leads to a great diversity of structures and structure–property relationships with potential for device applications. Exactly the same group theoretical constraints apply as for perovskite superstructures, and these determine the form of coupling of different order parameters with strain, permissible couplings between different order parameters and the full range of possible structures which might result.

The primary objective of the present paper is to present a group theoretical treatment of martensitic materials which can be derived from the simplest b.c.c. parent structure with space group 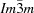. It has been notoriously difficult to distinguish between structure types on the basis of diffraction observations alone when the distinctions involve subtle differences in screw axes or glide planes. The software package *ISOTROPY* produces lists of allowable space groups which are definitive for subgroup structures and can be used to resolve such ambiguities. In addition, strain fields are long ranging so that the interaction length of the order parameter(s) is (are) also long ranging. As a consequence, critical fluctuations tend to be suppressed and the resulting changes in physical properties are expected to evolve according to mean field behaviour. Landau theory therefore provides a rigorous and quantitative framework for representing the thermodynamic and structural evolution of martensitic phases with single or multiple instabilities in response to changing temperature, pressure, stress, magnetic field and electric field. Finally, it is well understood that particular properties of interest can be engineered or tuned by changing other properties. In other words, one order parameter, such as for atomic ordering, can be adjusted to optimize the evolution of a second, such as magnetic moment, to produce, say, a desirable magnetocaloric response. These interactions will differ according to the form of allowed coupling between two (or more) order parameters, as λ*Q*_1_ *Q*_2_, λ*Q*_1_ *Q*_2_^2^, λ*Q*_1_^2^ *Q*_2_, λ*Q*_1_^2^ *Q*_2_^2^.

## 2. Group theoretical analysis   

### 2.1. Parent structures   

**Figure 1:**
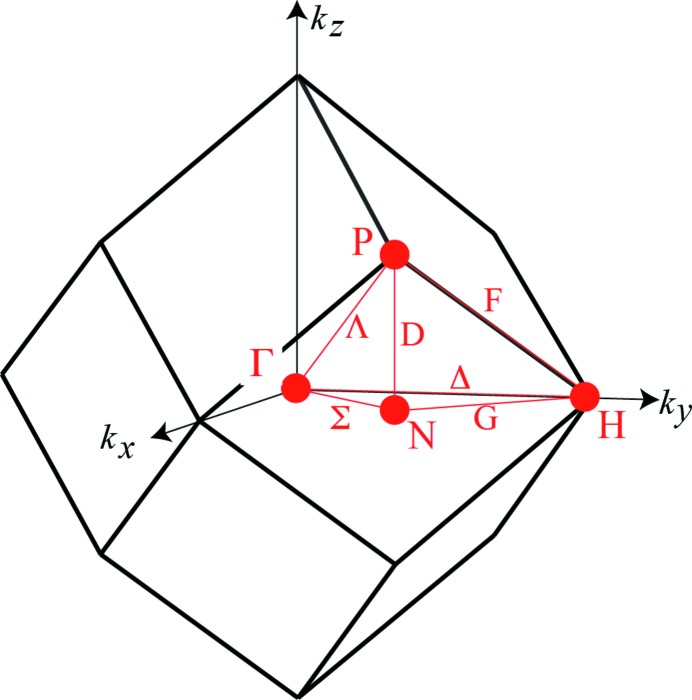
Brillouin zone for 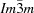 structures. Atomic ordering to give subgroup structures listed in Table 1 ▸ is based on order parameters belonging to irreducible representations (irreps) at special points H and P.

**Figure 2:**
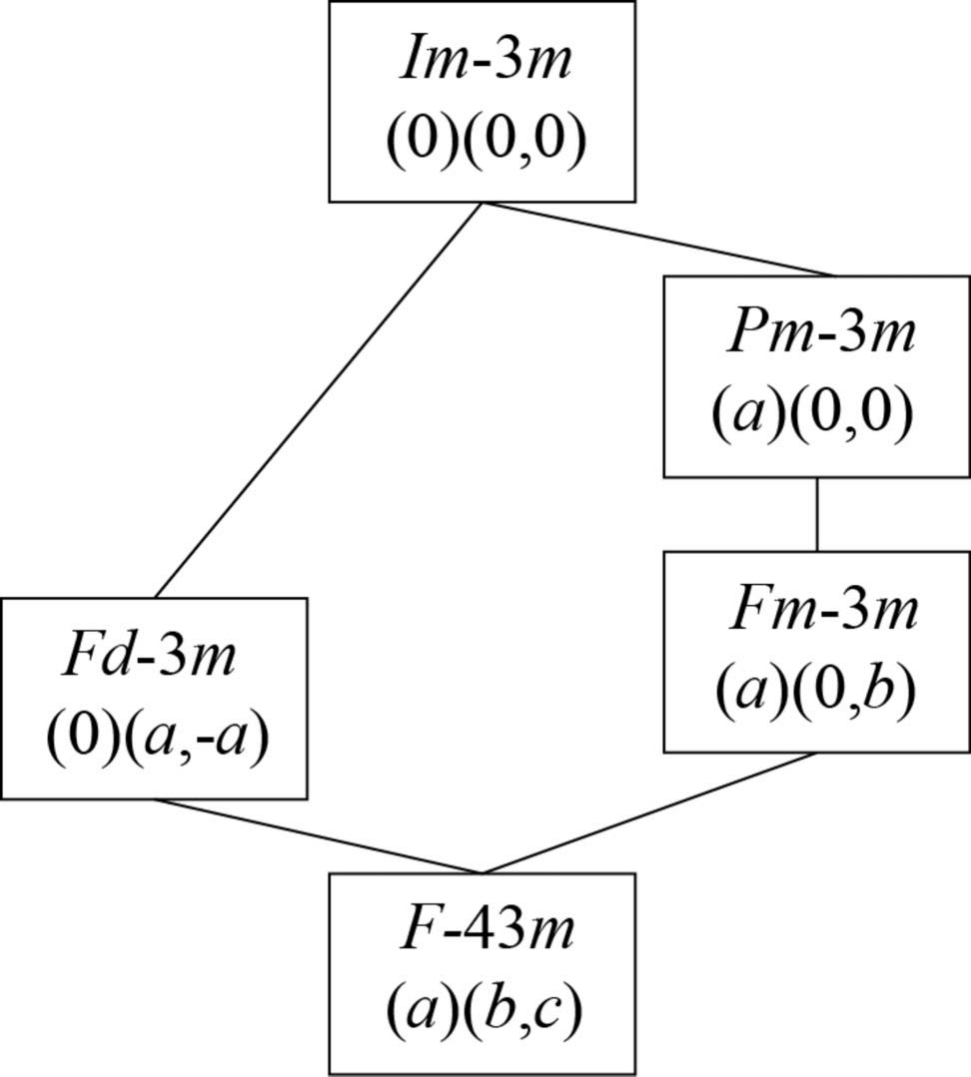
Hierarchy of ordered structures, as specified with respect to order parameters belonging to irreps 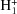 and P1. The transitions indicated by solid lines are allowed to be continuous according to Landau theory.

Table 1 ▸, after Graf *et al.* (2011 ▸), lists the generic stoichiometry and structures of Heusler-type phases (*XX*′*YZ*) which can be derived from a parent body-centred cubic (b.c.c.) structure. Here *X*, *X*′, *Y*, *Z* represent different elements that can combine together. Ordering of atoms according to order parameters with symmetry determined by irreducible representations of space group 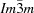 are also given [using the notation of Miller & Love (1967 ▸) here and throughout the rest of the paper]. These belong to the special points P, [1/2, 1/2, 1/2], and H, [0,1,0], of the Brillouin zone (Fig. 1 ▸), and give rise to four distinct subgroups. For example, the B2 structure of NiTi with space group 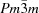 has a single nonzero order parameter with 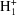 symmetry. The L2_1_ structure of Cu_2_MnAl, which is the classic *X*_2_ *YZ* Heusler structure, has space group 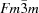 and two nonzero order parameter components, one with 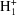 symmetry and the second with P1 symmetry. The DO_3_ structure of BiF_3_ is similar, where now *X* = *Y*. The different ordered structures form a hierarchy of subgroup structures from the 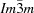 parent, as set out in Fig. 2 ▸. Solid lines in this figure represent phase transitions which are allowed by symmetry to be thermodynamically continuous according to Landau although, because they require rearrangement of atoms, would be expected to be slow.

### 2.2. Martensite structures   

The ferroelastic transitions which give rise to martensitic phases are characterized primarily by two effects, substantial shear strains and the development of large unit cells. Both depend on the symmetry of the driving order parameter(s) and their coupling with strain. Most of the observed product structures appear to be understandable in terms of separate order parameters which have symmetry properties related to the Brillouin zone centre (Γ point in Fig. 1 ▸) and points along one of the 〈110〉* directions of the reciprocal lattice for 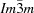 structures (Σ line of Fig. 1 ▸). These are set out in Table 2 ▸ for a single reference structure with space group 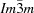 (the A2 structure in Table 1 ▸). If the transitions were driven solely by an electronic instability, such as band Jahn–Teller in Ni_2_MnGa (Fujii *et al.*, 1989 ▸; Brown *et al.*, 1999 ▸), the order parameter components would belong to irrep 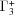 in most cases and the product structures would be tetragonal or orthorhombic. For example, 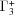 (*a*,0) would give structures with space groups *I*4/*mmm*, *P*4/*mmm*, *I*4_1_/*amd* or 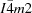, depending on the form of atomic order, and 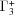 (*a*,*b*) would give corresponding orthorhombic structures (Table 2 ▸). A 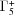 order parameter is also possible, however, and in the simplest cases would give orthorhombic structures with space groups *Fmmm*, *Cmmm*, *Immm*, *Imma* or *Imm*2 (Table 2 ▸).

By way of contrast, the driving mechanism for transitions with order parameters belonging to points along the Σ line is generally considered to involve an incipient soft mode [*e.g.* in Ni–Mn–Ga alloys (Stuhr *et al.*, 1997 ▸; Mañosa *et al.*, 2001 ▸; Moya *et al.*, 2006 ▸) and in Ti–Pd–Cr (Shapiro *et al.*, 2007 ▸)]. Observed repeats along [110]* of the reference 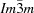 structure, varying between 2 and ∼14 (110) planes, correspond to **k** vectors for the active representation (*k*-active in Table 2 ▸) of between (1/2,1/2,0) and ∼(1/14,1/14,0); *k*-active = (1/2,1/2,0) corresponds to the N-point, [1/2, 1/2, 0], of the Brillouin zone for 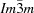 structures (Fig. 1 ▸). Taking 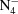 as the active representation leads to a variety of orthorhombic or monoclinic structures depending on whether the 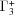 contribution is (*a*,0) or (*a*,*b*), respectively. Combining 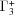
(*a*,0) and 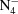 (0,0,0,0,*a*,0) leads to structures with space groups *Cmcm*, *Pmma*, *Pmmn*, *Pnma* and *Pmn*2_1_ as subgroups of 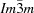, 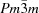, 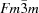, 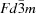 and 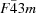, respectively. Combining 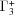
(*a*,*b*) with the simplest 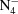 components gives monoclinic structures, *C*2/*m*, *P*2/*m*, *P*2_1_/*m*, *P*2_1_/*c*, *P*2_1_. Other combinations of nonzero components for 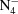 are possible and will lead to a wide variety of predicted structures, but reported structure types appear generally to require only one nonzero component.

Repeat distances along [110]* (with respect to the cubic *I* lattice)^1^ are observed to be incommensurate in some cases but are commonly referred to in terms of a commensurate repeat, *n*, such as 3, 5 and 7 for 3M, 5M and 7M structures, where *n* corresponds to the number of atomic layers parallel to (110) involved in a particular sequence of atomic displacements.The layers may be slightly displaced according to a conventional sinusoidal modulation or, as illustrated for example by Otsuka *et al.* (1993 ▸), displaced (shuffled) in consequence of the stacking characteristics of these nearly close-packed planes. In either case, we can describe the situation using irrep Σ_2_ at **k** vector (1/*n*,1/*n*,0) with just one component of the 12 component order parameter nonzero. The incommensurate case can be treated using the same 12 component Σ_2_ order parameter with just one nonzero component, by taking the **k** vector for the active representation to be (ξ,ξ,0). Otsuka *et al.* (1993 ▸) introduced a new description in which 3M, 5M and 7M were relabelled as 6M, 10M and 14M because they chose to describe the structures on centred unit cells. In the 5M/10M structure, for example, the (110) layers have a sequence of five shuffles that must occur twice in the unit cell to achieve a *B*-centred rather than primitive (in the case of a primitive starting structure) cell. An earlier nomenclature, for at least some of these martensites, is based on the number of (110) layers, in most cases a larger number, needed to complete a stacking sequence for these nearly close-packed atomic layers.

Considering the example of a parent structure with 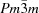 ordering (from Table 2 ▸, see also Table 3 ▸), the space group of the orthorhombic structure [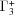
(*a*,0)] becomes *Amm*2 if *n* = 3, *Pmma* or *Pbam* if *n* = 4 and *Amm*2 if *n* = 5. For odd values of *n* the structures obtained are either orthorhombic on a cell in *Amm*2 comprising 2*n* layers, or monoclinic in *P*2/*m.* The monoclinic structures [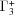
(*a*,*b*)] all have space group *P*2/*m*.

**Figure 3:**
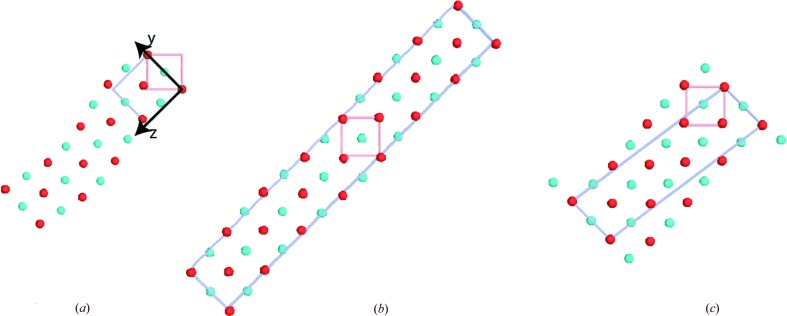
Examples of the graphics output from *ISODISTORT* (Stokes *et al.*, 2017 ▸). (*a*) An incommensurate modulation with **k** vector (0.143,0.143,0) applied to a parent structure in 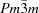 (*e.g.* NiTi, Ni red, Ti blue). The basic space group for the distorted structure is *Ammm*, and the figure shows, as well as the parent cell, the cell corresponding to this basic (average) symmetry. Note that the basic symmetry is orthorhombic. The modulation vector is along the *z* axis of the *Ammm* cell, and the period is 1/0.143, *i.e.* approximately seven (110) planes. (*b*) and (*c*) show results obtained from applying a commensurate modulation, **k** vector (1/7,1/7,0). It can be seen that, though the displacements have a period of seven (110) planes, the atomic arrangement precludes the construction of a simple unit cell with this period. The unit cell in (*b*) is obtained in orthorhombic symmetry, *Amm*2, by extending the cell to 14 (110) planes, and the unit cell in (*c*) by resorting to the monoclinic symmetry *P*2/*m*. The symmetries in (*b*) and (*c*), and especially the monoclinic symmetry in (*c*), may be artefacts arising from commensurate choices for the modulation vector **k**.

If the modulations are treated as incommensurate, the result is a structure in superspace group *Ammm*(0,0,γ)0*s*0 (Tables 2 ▸ and 3 ▸). This represents a structure (Fig. 3 ▸ *a*) with basic (average) orthorhombic symmetry *Ammm* and a modulation vector parallel to the *z* axis of the *Ammm* cell. The trailing 0*s*0 is to indicate that the second symmetry operator, the mirror plane perpendicular to the *y* axis, reverses the phase of the modulation. The lattice vectors and origin of this *Ammm* cell are given by the first three components of the four dimensional vectors shown in Tables 2 ▸ and 3 ▸. The repeat distance of the modulation will be 1/ξ. The variations in symmetry obtained with commensurate modulation vectors [Figs. 3 ▸(*b*) and 3 ▸(*c*)] may represent examples of the artefacts encountered when incommensurate modulations are approximated as commensurate (Janssen *et al.*, 2006 ▸).

For a structure with ordering on the basis of 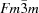, the orthorhombic product structures have space groups *Imm*2 (*n* = 3), *Pmma* (*n* = 4), *Imm*2 (*n* = 5), and the monoclinic structures have space group *P*2/*m* (*n* = even) or *C*2/*m* (*n* = odd). Comparison of these with known structures needs to take account of the fact that the values of *n* in Table 2 ▸ refer to 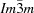 as the parent structure. The 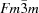 structure has a unit cell which is double the dimensions of the 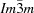 cell, so that *n*_I_ = 6 (**k** = 1/6,1/6,0) with respect to the latter becomes *n*_F_ = 3 (**k** = 1/3,1/3,0) with respect to the former. The *Pmma* structure reported by Brown *et al.* (2006 ▸) as the product of a phase transition from a parent structure with space group 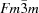 has *n*_F_ = 2 (**k** = 1/2,1/2,0), and would correspond to the structure with *n*_I_ = 4 (**k** = 1/4,1/4,0) in Table 2 ▸. The *Pnnm* structure with *n*_F_ = 3 reported by Brown *et al.* (2002 ▸) would correspond to the structure with *n*_I_ = 6 (**k** = 1/6,1/6,0) in Table 2 ▸, and similarly for *n*_F_ = 7, *n*_I_ = 14. The *P*2/*m* structure described by Brown *et al.* (2011 ▸) has *n*_F_ = 3, **k** = (1/3,1/3,0) and corresponds to the structure with *n*_I_ = 6, **k** = (1/6,1/6,0) in Table 2 ▸.

Table 3 ▸ contains the same information as Table 2 ▸ for the specific case of a 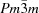 parent structure, in a slightly different format that might prove to be more practicable when considering B2 structures such as NiTi and NiAl or TiAl and RuNb. The zone boundary irrep 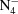 becomes 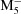 so that the structural relationships acquire the more familiar form for 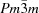, *Pmma* and *P*2_1_/*m* structures as already set out by Barsch (2000 ▸). 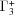
(*a*,0) gives *P*4/*mmm*, corresponding to the β′ structure of RuNb stable between ∼1030 and ∼1170 K (*e.g.* Dirand *et al.*, 2012 ▸; Nó *et al.*, 2015*a* ▸, 2015*b* ▸), the room-temperature structure of TiAl (Duarte *et al.*, 2012 ▸) and the structure of Ni*_x_*Al_1−*x*_, *x* ≃ 0.64, quenched from high temperatures (Potapov *et al.*, 1997 ▸). The low-temperature (β′′) structure of RuNb has been reported to be either ortho­rhombic, *Cmmm* (Chen & Franzen, 1989 ▸), or monoclinic, *P*2/*m* (Nó *et al.*, 2015*a* ▸,*b* ▸) or *P*2_1_/*m* (Mousa *et al.*, 2009 ▸). All three of these structure types would have the same unit cell as some permutation of 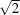 *a*_o_ × 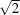 *a*_o_ × *a*_o_, but differing in the combination of driving order parameters.

Other sets of structures can be generated by considering *k*-active as having directions along several of the 〈110〉^*^ directions, instead of just one. For example, if there are three equivalent directions, (1/3,1/3,0), (1/3,0,1/3), (0,1/3,−1/3), a trigonal structure is obtained from a 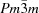 parent. This is the R-phase observed in Ni–Ti and Au–Cd alloys (*e.g.* Otsuka & Ren, 2005 ▸; Zolotukin *et al.*, 2012 ▸), and can be generated with (*a*,0,0,0,*a*,0,0,0,0,0,*a*,0) as components of the Σ_2_ order parameter (Table 3 ▸). As reviewed by Otsuka & Ren (2005 ▸), various suggestions have been made for the correct space group of this structure, including 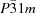 (Vatanayon & Hehemann, 1975 ▸; Goo & Sinclair, 1985 ▸), *P*3 (Ohba *et al.*, 1992 ▸; Hara *et al.*, 1997 ▸) and 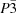 (Schryvers & Potapov, 2002 ▸; Sitepu, 2003 ▸). The group theoretical treatment set out here gives space group 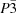 for the particular combination of order parameters listed in Table 3 ▸. If there are just two equivalent directions, (1/3,1/3,0), (1/3,0,1/3), tetragonal structures will result, but these have not been explored further.

For practical convenience when considering L2_1_ Heusler compounds, Table 4 ▸ shows subgroup structures with respect to 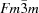, rather than 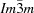, as the parent structure. This includes, for example, the martensite structures of Ni_2_Mn_1.44_Sn_0.56_ and Ni_2_Mn_1.48_Sb_0.52_ described by Brown *et al.* (2006 ▸, 2010 ▸), which have space group *Pmma* and, when referring to the larger parent cell, *k*-active = (1/2,1/2,0). Ni_2_MnGa has two martensitic structures with space group *Pnnm*: *k*-active = (1/3,1/3,0) and (1/7,1/7,0) (Brown *et al.*, 2002 ▸). The martensite structure of Ni_1.84_Mn_1.64_In_0.52_ has space group *P*2/*m* and *k*-active = (0,0,0) and (1/3,1/3,0) (Brown *et al.* 2011 ▸). The room-temperature structure of Ni_2.19_Mn_0.82_Ga has space group *I*4/*mmm* (Banik *et al.*, 2007 ▸), which corresponds to 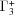
(*a*,0), 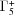
(0,0,0), Σ_2_ (0,0,0,0,0,0,0,0,0,0,0,0), *k*-active = (0,0,0). A limitation of using subgroups of 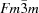 in terms of a sequence as 1/*n*, *n* = 2, 3, 4…, however, is that the *n* = odd entries in Table 2 ▸ are not included. For example, a structure with **k** = (1/3,1/3,0) in Table 2 ▸ would have *k*-active = (2/3,2/3,0) if it was added to Table 4 ▸. The choice of label, 3M or 6M, 5M or 10M, *etc*., also depends on whether reference is being made to the superlattice repeat, with respect to the 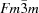 cell, or to the number of atomic layers in the repeating unit (Singh *et al.* 2015 ▸).

### 2.3. Primary and secondary order parameters   

Inspection of Table 2 ▸ reveals that the 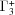 order parameter can act on its own, whereas nonzero values of components of 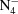 and Σ_2_ are always accompanied by nonzero values of components from both 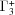 and 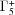. The latter can just be secondary order parameters, consequential on coupling to tetragonal and orthorhombic shear strains, *e*_t_ and *e*_o_ (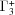), or shear strains *e*_4_, *e*_5_, *e*_6_ (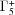), but they could also represent primary order parameters due to separate instabilities. Similarly, 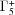 is invariably accompanied by nonzero values of components of 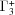 which may be secondary but could be primary from a separate, additional instability. At the heart of the diversity of martensite structures is the existence of both the fundamental electronic instability and the possibility of additional instabilities associated, for example, with the soft mode.

If the 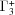 order parameter acts alone, the pattern of spontaneous strains is determined by coupling terms in the Landau free-energy expansion
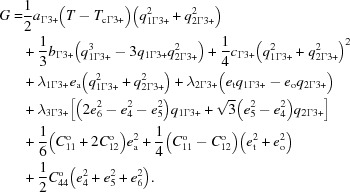
Here *q* represents order parameter components, *a*, *b*, *c* are standard Landau coefficients, λ’s are coupling coefficients, 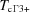 is the critical temperature, *e*_a_ (= *e*_1_ + *e*_2_ + *e*_3_) is the volume strain, *e*_t_ [= (2*e*_3_ − *e*_1_ −*e*_2_)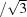] is the tetragonal shear strain, *e*_o_ (= *e*_1_ − *e*_2_) is the orthorhombic shear strain, *e*_4_, *e*_5_ and *e*_6_ are the remaining shear strains, and 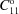, 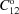, 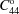 are elastic constants of the parent cubic structure. If the 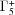 order parameter acts alone, the Landau expansion is
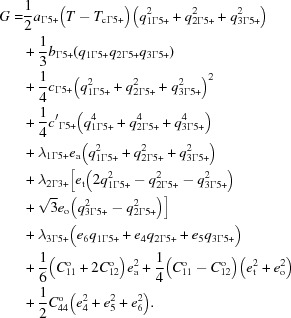
If the single order parameter is 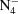 or Σ_2_, the equivalent Landau expansion requires six or 12 components, respectively, though the space groups of real structures so far identified can be understood with just one or two nonzero values. The generality of couplings with strain is that they must be linear–quadratic, 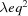, or biquadratic, 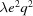. For each of the three cases, the relationship(s) between individual strains and the driving order parameter(s) can be found by applying the equilibrium condition, ∂*G*/∂*e* = 0, in the usual way (*e.g.* Carpenter *et al.*, 1998 ▸).

In materials with multiple instabilities, coupling between the separate order parameters can be direct or indirect *via* the common strain. The simplest generalization here is for coupling between a zone centre order parameter, *q*_Γ_, and an order parameter from along the Σ line out to the N point, *q*_Σ_. Biquadratic coupling, 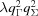, is always allowed between two order parameters with different symmetries and a wide variety of sequences of structures and phase transitions can result (Salje & Devarajan, 1986 ▸). The important parameters are the strength of coupling, λ, and the relative critical temperatures of the two instabilities, *T*_cΓ_ and *T*_cΣ_. Linear–quadratic coupling, 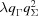, is also allowed for some combinations, but leads to a much more restricted range of possibilities (Salje & Carpenter, 2011 ▸). In principle, *T*_cΣ_ > *T*_cΓ_ would be expected to give rise to a single transition from a state with *q*_Γ_ = 0, *q*_Σ_ = 0 to one with *q*_Γ_ ≠ 0, *q*_Σ_ ≠ 0 because *q*_Σ_ generates a conjugate field for *q*_Γ_. Alternatively, for *T*_cΣ_ < *T*_cΓ_, the sequence can be a second-order transition to a structure with *q*_Γ_ ≠ 0, *q*_Σ_ = 0, followed by a first-order transition to a phase with *q*_Γ_ ≠ 0, *q*_Σ_ ≠ 0. Coupling terms between 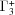 and 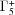 can in principle also be linear–quadratic and biquadratic as:
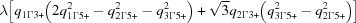
and
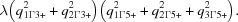
Indirect coupling *via* shear strains would give the linear–quadratic term while coupling *via* the volume strain would give rise to the biquadratic term.

An example of coupling between order parameters for instabilities with two nonzero components of 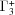 and one nonzero component of 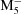, with respect to a parent 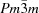 structure, can be represented by the Landau expansion:
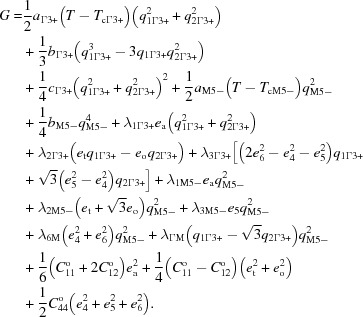
From Table 3 ▸, if the nonzero components of 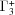 are (*a*, 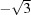 *a*) and the nonzero components of 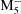 are (0,0,*c*,*c*,0,0), the resultant structure has *Pmma* symmetry (B19 structure). This has 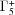 (0,0,*b*), *i.e.* the shear strain *e*_5_, as a secondary order parameter. However, the same outcome could be obtained using 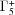 with 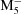 as primaries and 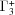 as secondary, or taking 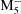 as driving and both 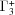 and 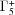 as secondary.

Treatment of magnetic transitions is beyond the scope of the present work but all the same symmetry and strain coupling arguments would apply. The only fundamental difference is that the coupling of a magnetic order parameter *M* with strains *e* will be of the form λ*eM* ^2^ or λ*e* ^2^ *M* ^2^. It follows that pseudoproper ferroelastic softening will not be observed if the transition is driven by the magnetic instability. A Landau expansion which includes strain as a driving order parameter, an order parameter for the structural modulations and the magnetic order parameter has been given by Vasil’ev *et al.* (2003 ▸). A simpler form, with only the Γ-point and magnetic order parameters, is given in Vasil’ev *et al.* (1999 ▸).

## 3. Some examples of real materials   

Applications of the group theoretical approach set out above can be illustrated with three specific examples, using alloys relating to NiTi, TiPd and Ni_2_MnGa.

### 3.1. NiTi, RuNb   

NiTi undergoes a single transition from the B2 structure to the B19′ structure at ∼335 K, corresponding to 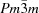
–*P*2_1_/*m* (Otsuka & Ren, 2005 ▸). *P*2_1_/*m* is not a symmetry subgroup of order 2 with respect to 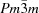, however, but it is a subgroup order 2 with respect to *Pmma*. Following Barsch (2000 ▸) and Otsuka & Ren (2005 ▸), there appear to be two instabilities and these are seen in sequence as 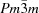
–*Pmma*–*P*2_1_/*m* in Ti_50_Ni_50−*x*_Cu*_x_* (Nam *et al.*, 1990 ▸). Symmetry relationships are as listed in Table 3 ▸: the active representations are 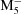 of 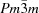 and 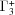 of *Pmma* (Barsch, 2000 ▸). With respect to 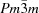 symmetry the two discrete electronic instabilities relate essentially to 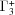 and 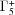, coupled to the M-point (zone boundary) mode.

Michal & Sinclair (1981 ▸) have given *a* = 2.885, *b* = 4.120, *c* = 4.622 Å, β = 96.8° for the unit cell of the monoclinic structure at room temperature, which corresponds to ∼*a*_o_ × 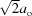 × 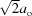, where *a*_o_ is the dimension of the primitive parent cubic structure. Using an orthogonal reference system with *X*, *Y* and *Z* parallel to crystallographic *x*, *y* and *z* of the parent structure, the nonzero shear strains are *e*_ty_ = (2*e*_2_ − *e*_1_ − *e*_3_)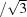, *e*_6_ = *e*_4_ ≠ *e*_5_. Here *e*_ty_ is the tetragonal shear strain with the unique axis aligned parallel to the crystallographic *y*-axis. In terms of the lattice parameters of the monoclinic structure, individual strains are given by *e*_2_ = (*a* − *a*_o_)/*a*_o_, *e*_1_ + *e*_3_ = 
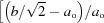
 + 
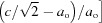
, *e*_5_ = 
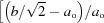
 
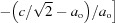
, *e*_4_ = *e*_6_ ≃ 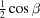. Using *a*_o_ as approximated by (*abc*/2)^1/3^, gives the values *e*_ty_ = −0.079, ∣*e*_5_∣ = 0.118, *e*_6_ = *e*_4_ ≃ −0.059. These three shear strains are substantially greater than any that are typically associated with transitions driven by phonon-related instabilities.

Evidence for a separate soft-mode transition in Ni–Ti alloys is revealed by the changes in transition sequences induced by addition of minor components in solid solution. The transition sequence in Ti_50_Ni_50−*x*_Fe*_x_* is 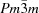
–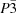
–*P*2_1_/*m* (B2–R–B19′) (Honma *et al.*, 1980 ▸), taking the R-phase as having space group 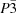. In a sample with *x* = 3.2, a precursor is incommensurate but the R-phase itself is commensurate (Shapiro *et al.*, 1984 ▸; Salamon *et al.*, 1985 ▸). There is a small discontinuity in the pseudocubic lattice angle, α, at the 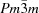
–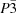 transition and this angle decreases to 89.3° with falling temperature (Salamon *et al.*, 1985 ▸). The transition is thus weakly first order, with the symmetry-breaking shear strain, *e*_4_ = *e*_5_ = *e*_6_ ≃ cosα, reaching a maximum value of ∼0.012, consistent with the transition being driven by softening of an acoustic phonon along the [110]^*^ branch (Satija *et al.*, 1984 ▸; Moine *et al.*, 1984 ▸). Salje *et al.* (2008 ▸) found the same strain variation in a different sample with the same composition. The electronic and soft-mode instabilities are suppressed to different extents with increasing Fe-content such that the stability field of the R-phase expands. In principle they could combine to produce superlattice structures with commensurate or incommensurate repeat distances along [110]^*^ but, for stoichiometric Ni–Ti, the lowest energy (*P*2_1_/*m*) structure is not a subgroup of 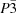 and has the two gamma point order parameters combined with an M-point order parameter. Parlinski & Parlinska-Wojtan (2002 ▸) have shown that the latter can also be understood in terms of a soft mode.

In NiTi, the 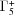 order parameter changes from (0,0,*b*) to (*b*,*b*,*c*) causing *Pmma* to become *P*2_1_/*m*. The same order parameter could be primary for the second symmetry change in RuNb where the sequence is 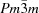
–*P*4/*mmm* [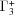 (*a*,0), 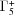 (0,0,0), 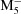 (0,0,0,0,0,0)]–*Cmmm* [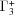 (*a*,0), 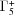 (*b*,0,0), 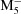 (0,0,0,0,0,0)] or *P*2/*m* [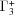 (*a*,*b*), 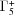 (0,0,*c*), 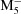 (0,0,*d*,*e*,0,0)]. The tetragonal shear strain, *e*_tz_ [= (2*e*_3_ − *e*_1_ − *e*_2_)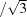], calculated from the lattice parameters given by Shapiro *et al.* (2006 ▸) for the tetragonal phase at 900 K, is 0.07 [*e*_1_ = (*a* − *a*_o_)/*a*_o_, *e*_2_ = (*b* − *a*_o_)/*a*_o_, *e*_3_ = (*c* − *a*_o_)/*a*_o_]. Tetragonal, *e*_tz_, and orthorhombic, *e*_o_ = *e*_1_ − *e*_2_, strains calculated from the orthorhombic lattice parameters given for 873 K are 0.14 and −0.02, respectively. In both cases, the same procedure as described above was used for estimating *a*_o_. The large increase in shear strain at the second transition is consistent with an electronic driving mechanism and 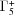 being primary.

### 3.2. Ni_2+*x*_Mn_1−*x*_Ga   

The L2_1_ Heusler compound Ni_2_MnGa is cubic, space group 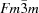, at high temperatures. Lowering of the symmetry from a parent 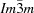 structure in which the atoms would be disordered between all the crystallographic sites is described by two order parameters, one belonging to irrep 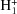 and the second to irrep P1. It undergoes two phase transitions during cooling, at ∼260 and ∼200 K. Following Brown *et al.* (2002 ▸), the first is to a ‘pre-martensitic’ structure which is incommensurate (Singh *et al.*, 2015 ▸) but can be represented in terms of an orthorhombic structure with space group *Pnnm* and unit cell *a* ∼ 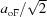, *b* ∼ 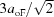, *c* ∼ *a*_oF_, where *a*_oF_ is the lattice parameter of the parent cubic F unit cell (Table 4 ▸; Brown *et al.*, 2002 ▸). The driving mechanism is related to softening of the (Σ_2_) soft acoustic phonon at **q** ∼ (1/3,1/3,0) (Zheludev *et al.*, 1995 ▸; Stuhr *et al.*, 1997 ▸; Mañosa *et al.*, 2001 ▸). Strains accompanying this transition are such that distortion from cubic lattice geometry is small (Brown *et al.*, 2002 ▸; Ohba *et al.*, 2005 ▸). Ohba *et al.* (2005 ▸) gave lattice parameters at 250 K as *a* = 5.8285, *b* = 5.8142, *c* = 5.7886 Å, which yield linear strain components *e*_1_ = (*a* − *a*_oF_)/*a*_oF_ = 0.003, *e*_2_ = (*b* − *a*_oF_)/*a*_oF_ = 0.001, *e*_3_ = (*c* − *a*_oF_)/*a*_oF_ = −0.004 (with the usual approximation for *a*_o_). Expressed in symmetry-adapted forms the tetragonal and orthorhombic shear strains are *e*_tz_ = −0.007 and *e*_o_ = 0.002, respectively.

The second transition is to a structure which may also be incommensurate but can be represented as being orthorhombic in the same space group, *Pnnm*, with unit cell *a* ∼ 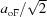, *b* ∼ 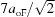, *c* ∼ *a*_oF_ (Table 4 ▸; Brown *et al.*, 2002 ▸; Ranjan *et al.*, 2006 ▸; Righi *et al.*, 2006 ▸; Zheludev *et al.*, 1996 ▸). Determining strains in the same way from the lattice parameters given by Brown *et al.* (2002 ▸), *a* = 4.2152, *b* = 29.3016, *c* = 5.5570 Å, gives *e*_t_ = −0.076 and *e*_o_ = 0.007, respectively. The factor of 10 increase in *e*_t_ with respect to the pre-martensitic phase seems to be characteristic for strain coupling with the 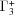 order parameter at a band Jahn–Teller transition. The two order parameters produce a large tetragonal strain from the electronic instability and multiplication of the cell dimension from the soft mode. There is also a nonzero order parameter component (*a*,0,0) belonging to 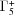 (Table 4 ▸), but it does not appear to drive any of the instabilities and is therefore genuinely secondary.

**Figure 4:**
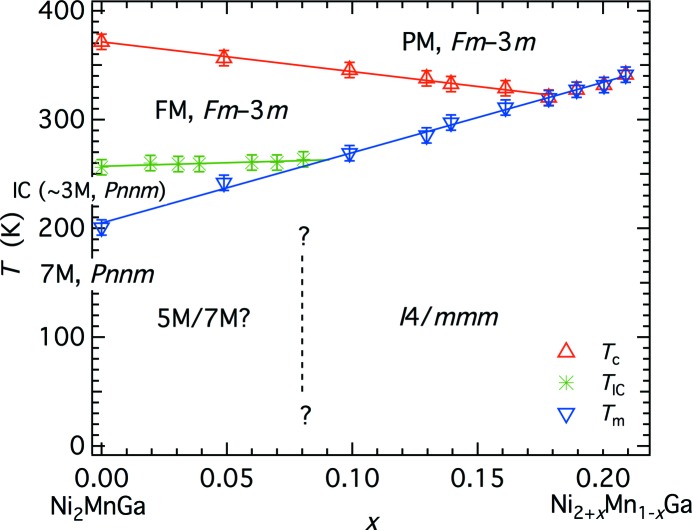
Mn-rich portion of the Ni_2+*x*_Mn_1−*x*_Ga phase diagram, after Vasil’ev *et al.* (2003 ▸) and Entel *et al.* (2014 ▸). An approximate location for the boundary between *Pnnm* structures (*q*_Γ3+_ ≠ 0, *q*_Σ2_ ≠ 0) and the *I*4/*mmm* structure (*q*_Γ3+_ ≠ 0, *q*_Σ2_ = 0) is based on the data given by Banik *et al.* (2007 ▸, their Table 1). *T*_c_ marks the paramagnetic (PM) to ferromagnetic (FM) transition.

Increasing the Ni content at the expense of Mn in Ni_2+*x*_Mn_1−*x*_Ga causes the transition temperatures for both transitions to increase, with slopes that give a diminishing field for the pre-martensite structure (Fig. 4 ▸, after Vasil’ev *et al.*, 2003 ▸; Entel *et al.*, 2014 ▸). The martensite structures also change from a 5M (*k*-active = (1/5,1/5,0) structure reported at *x* = 0.02 (Vasil’ev *et al.*, 2003 ▸) to 7M (*k*-active = (1/7,1/7,0) and then to the *I*4/*mmm* structure, which has the (*a*,0) electronic distortion only. Linear-quadratic coupling, 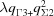, is permitted by symmetry and, from the discussion in §2.3 above, would be expected to give rise to a single transition directly from a state with 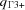 = 0, *q*_Σ2_ = 0 to one with 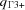 ≠ 0, *q*_Σ2_ ≠ 0 for *T*_cΣ2_> *T*_cΓ3+_. Instead this sequence is observed at relatively high Ni contents where *T*_cΣ2_ falls below the martensitic transition temperature. The implication is that linear–quadratic coupling is not a dominant factor in determining the stability of the martensitic structures. Either coupling between the two order parameters is weak or it is dominated by biquadratic terms, 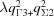, which could arise *via* the common volume strain. The *q*_Σ2_ component presumably diminishes with increasing Ni-content since it is zero in the *I*4/*mmm* structure.

### 3.3. Ti_50_Pd_50−*x*_Cr_*x*_   

**Figure 5:**
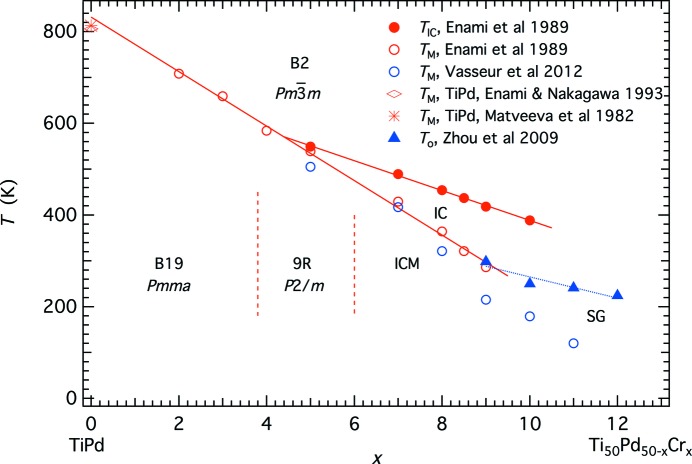
Simplified phase diagram showing the variation of transition temperatures for B2–incommensurate (IC) and B2–B19, IC–9R, IC–incommensurate martensite (ICM) transitions at the Pd-rich end of the TiPd–TiCr solid solution. The first-order martensitic transition occurs in stoichiometric TiPd at ∼810 K (Matveeva *et al.*, 1982 ▸; Enami & Nakagawa *et al.*, 1993 ▸). Vertical dashed lines are approximate composition limits for different martensitic phases observed at room temperature, based on observations of Enami *et al.* (1989 ▸) and Schwartz *et al.* (1995 ▸).

Ti_50_Pd_50−*x*_Cr*_x_* represents a further example of changing structural sequences with increasing doping. There is a crossover between two sequences, 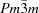 (B2)–*Pmma* (B19) and 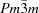
–incommensurate (IC)–incommensurate martensite (ICM), at *x* ∼ 4.5 (Fig. 5 ▸, following Enami *et al.*, 1989 ▸; Schwartz *et al.*, 1995 ▸; Shapiro *et al.*, 2007 ▸). In contrast with Ni_2+*x*_Mn_1−*x*_Ga, the trend is of decreasing transition temperatures with increasing doping, and structures with *q*_Σ2_ ≠ 0 appear at relatively high values of *x*. The 9R structure is monoclinic (*P*2/*m*) and has a Σ_2_ repeat of three, while the ICM structure has IC repeat distances derived from the Σ_2_ order parameter over a range between ∼3 and ∼5. This pattern is similar to that of other Ti–Pd alloys with V, Mn, Fe, Ce or Ni as the additional, minor component (Enami & Nakagawa, 1993 ▸).

Linear-quadratic coupling, 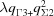 is again allowed by symmetry but the transition sequences with falling temperature are the same as observed for Ni_2+*x*_Mn_1−*x*_Ga in not complying with what would be expected from the generalized treatment of Salje & Carpenter (2011 ▸). In this system, the contributions of *q*_Σ2_ clearly increase with increasing Cr content as the transition temperature for structures with *q*_Γ3+_ ≠ 0 reduces. Other martensite materials with group–subgroup relationships need to be examined, but it appears that biquadratic coupling may be dominant in systems with band Jahn–Teller transitions.

## 4. Patterns of elastic anomalies due to strain–order parameter coupling   

Differences in the symmetry properties of martensitic structures define distinct patterns of thermodynamic behaviour and are not simply matters of form or representation. The most obvious way to distinguish between them is by observing variations in the elastic constants, as set out more generally, for example, by Carpenter & Salje (1998 ▸). Due to bilinear coupling of a symmetry breaking shear strain with the primary order parameter, λ*e*_sb_ *q*, transitions driven by the 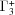 order parameter will show pseudoproper ferroelastic softening of *C*_11_–*C*_12_ and those driven by 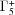 will show pseudoproper ferroelastic softening of *C*_44_ as temperature reduces towards the transition point. Transitions driven by a Σ_2_ (or 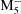) order parameter will be improper ferroelastic with stepwise softening in either or both of *C*_11_–*C*_12_ and *C*_44_ below the transition point due to coupling of the form λ*e*_sb_ *q* ^2^.

In some previous Landau expansions produced to describe the electronic and soft mode instabilities with order parameters belonging separately to zone centre and zone boundary irreps, strain itself was used as the driving order parameter for the electronic part (*e.g.* Entel *et al.*, 2006 ▸; Vasil’ev *et al.*, 2003 ▸). In other words, the expectation was for a true-proper, as opposed to pseudo-proper, ferroelastic transition, with specific implications for the evolution of the elastic constants (*e.g.* Carpenter & Salje, 1998 ▸). The pattern of evolution of the shear modulus, at least, for the simplest case of the 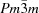
–*P*4/*mmm* transition in Ru–Nb, which involves only the 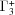 order parameter, is of nonlinear softening as the transition point is approached from both sides (Dirand *et al.*, 2012 ▸; Nó *et al.*, 2015*a* ▸,*b* ▸). This fits with pseudoproper behaviour which, in turn, suggests that it is the change in electronic structure and not the strain that provides the driving order parameter.

The compilation of temperature-dependent single-crystal elastic constants given by Otsuka & Ren (2005 ▸, their Fig. 38) for Ni–Ti–Fe and Ni–Ti–Cu alloys shows softening of both *C*_11_–*C*_12_ and *C*_44_ as the martensitic transitions are approached from above. This confirms the proximity of electronic instabilities with symmetries belonging to both 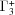 and 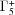.

The pattern of evolution of both *C*_11_–*C*_12_ and *C*_44_ in Ni_2_MnGa ahead of and through the L2_1_ (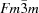) to IC (∼3M, *Pnnm*) transition (*e.g.* Mañosa *et al.*, 1997 ▸; Stipcich *et al.*, 2004 ▸) is characteristic of improper ferroelastic behaviour, implying that the driving order parameter relates predominantly to Σ_2_ and, hence, that 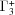 is secondary. Some precursor softening of *C*_11_–*C*_12_ has been reported by Stipcich *et al.* (2004 ▸), however, and this was enhanced following heat treatments (Seiner *et al.*, 2013 ▸). A driving role clearly can exist for 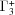 but with a strength that depends on the structural state of the sample. The additional factor controlling this strength is most likely the degree of atomic order, as could be expressed in terms of coupling of Σ_2_ and 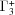 order parameters with 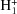 and P1 order parameters. This coupling is biquadratic in lowest order, 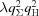, 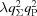, 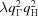, 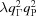. As a consequence, the effects of changes in the degree of atomic order are most likely to be seen as renormalization of the critical temperature for the martensitic and soft-mode transitions. This is exactly analogous to the influence of Fe/Mo ordering on phase transitions in Sr_2_FeMoO_6_ (Yang *et al.*, 2016 ▸).

## 5. Conclusions   

Group theoretical analysis of order parameters relating to atomic ordering, electronic instabilities and soft-mode behaviour has been used to specify the symmetry relationships which can lead to a wide variety of structures in alloys with multiple premartensitic and martensitic phase transitions.

Coupling between order parameters can be direct or indirect *via* coupling with common strains. The most significant coupling in this context is between Γ-point and Σ_2_ order parameters, with both linear–quadratic and biquadratic terms allowed. In the small number of materials considered as examples here, the characteristic sequences of transformations expected from linear–quadratic coupling are not observed, however.

Transformation sequences and phase stabilities in a given material depend on the balance of energies associated with each of the possible order parameters. The composition and degree of atomic order can be chosen so that, in principle, the different order parameters and the strength of their coupling can be engineered to produce optimal properties in functional materials.

In terms of testing models of multiple phase transitions in martensitic phases, observed patterns of elastic constants are likely to prove definitive, because of the characteristic patterns of elastic softening and stiffening in ferroelastic materials due to bilinear, linear–quadratic and biquadratic coupling with strains.

## Figures and Tables

**Table 1:** Derivative structures based on a body centred cubic parent structure with space group 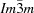 (after Graf *et al.*, 2011 ▸) Nonzero order parameters for irreducible representations 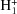 and P1 of 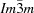 describe the atomic ordering schemes in each case. *Z* in the 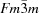 structures (*e.g.* Bi in BiF_3_) is taken to be on Wyckoff *a*. *X*, *X*′, *Y*, *Z* represent different possible combinations of elements.

Generic chemical components	Generic chemical formula	Example	Conventional label	Space group	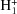	P1	Unit-cell edge with respect to 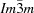
*X* = *X*′ = *Y* = *Z*	*X*_4_	W	A2	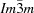	(0)	(0,0)	*a*_o_
*X* = *X*′, *Y* = *Z*	*X*_2_ *Y*_2_	NiTi	B2	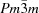	(*a*)	(0,0)	*a*_o_
*X* = *X*′, *Y*, *Z*	*X*_2_ *YZ*	Cu_2_MnAl	L2_1_	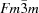	(*a*)	(0,*b*)	2*a*_o_
*X* = *X*′ = *Y*, *Z*	*X*_3_ *Z*	BiF_3_	DO_3_	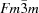	(*a*)	(0,*b*)	2*a*_o_
*X* = *Y*, *X*′ = *Z*	*X*_2_ *X*′_2_	NaTl	B32a	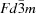	(0)	(*a*,−*a*)	2*a*_o_
*X*, *X*′ = *Y*, *Z*	*XX*′_2_ *Z*	CuHg_2_Ti	*X*	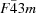	(*a*)	(*b*,*c*)	2*a*_o_
*X*, *X*′, *Y*, *Z*	*XX*′*YZ*	LiMgPdSn	*Y*	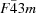	(*a*)	(*b*,*c*)	2*a*_o_

**Table 2:** Symmetry relationships, order parameters and unit-cell configurations for selected subgroups of space group 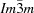, as derived using the group theory program *ISOTROPY* (Stokes *et al.*, 2007 ▸) Two orientations have been given in some cases for 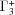, (*a*,0) and (*a*,*a* 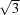), to illustrate how this choice affects basis vectors which define the unit cell of the subgroup structure.

Space group	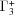	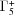	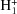	P1		Lattice vectors	Origin
229 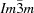	(0,0)	(0,0,0)	(0)	(0,0)		(1,0,0),(0,1,0),(0,0,1)	(0,0,0)
139 *I*4/*mmm*	(*a*,0)	(0,0,0)	(0)	(0,0)		(1,0,0),(0,1,0),(0,0,1)	(0,0,0)
71 *Immm*	(*a*,*b*)	(0,0,0)	(0)	(0,0)		(1,0,0),(0,1,0),(0,0,1)	(0,0,0)
69 *Fmmm*	(*a*,0)	(*b*,0,0)	(0)	(0,0)		(1,1,0),(0,0,1),(1,−1,0)	(0,0,0)
							
221 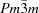	(0,0)	(0,0,0)	(*a*)	(0,0)		(1,0,0),(0,1,0),(0,0,1)	(0,0,0)
123 *P*4/*mmm*	(*a*,0)	(0,0,0)	(*b*)	(0,0)		(1,0,0),(0,1,0),(0,0,1)	(0,0,0)
47 *Pmmm*	(*a*,*b*)	(0,0,0)	(*c*)	(0,0)		(1,0,0),(0,1,0),(0,0,1)	(0,0,0)
65 *Cmmm*	(*a*,0)	(*b*,0,0)	(*c*)	(0,0)		(1,−1,0),(1,1,0),(0,0,1)	(0,0,0)
							
225 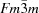	(0,0)	(0,0,0)	(*a*)	(0,*b*)		(2,0,0),(0,2,0),(0,0,2)	(1/2,1/2,1/2)
139 *I*4/*mmm*	(*a*,0)	(0,0,0)	(*b*)	(0,*c*)		(1,1,0),(−1,1,0),(0,0,2)	(1/2,1/2,1/2)
69 *Fmmm*	(*a*,*b*)	(0,0,0)	(*c*)	(0,*d*)		(2,0,0),(0,2,0),(0,0,2)	(1/2,1/2,1/2)
71 *Immm*	(*a*,0)	(*b*,0,0)	(*c*)	(0,*d*)		(1,1,0),(−1,1,0),(0,0,2)	(1/2,1/2,1/2)
							
227 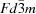	(0,0)	(0,0,0)	(0)	(*a*,−*a*)		(2,0,0),(0,2,0),(0,0,2)	(3/4,3/4,3/4)
141 *I*4_1_/*amd*	(*a*,0)	(0,0,0)	(0)	(*b*,−*b*)		(−1,1,0),(−1,−1,0),(0,0,2)	(3/4,3/4,3/4)
70 *Fddd*	(*a*,*b*)	(0,0,0)	(0)	(*c*,−*c*)		(2,0,0),(0,2,0),(0,0,2)	(3/4,3/4,3/4)
74 *Imma*	(*a*,0)	(*b*,0,0)	(0)	(*c*,−*c*)		(1,−1,0),(1,1,0),(0,0,2)	(3/4,3/4,3/4)
							
216 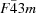	(0,0)	(0,0,0)	(*a*)	(*b*,*c*)		(2,0,0),(0,2,0),(0,0,2)	(0,0,0)
119 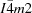	(*a*,0)	(0,0,0)	(*b*)	(*c*,*d*)		(1,−1,0),(1,1,0),(0,0,2)	(0,0,0)
22 *F*222	(*a*,*b*)	(0,0,0)	(*c*)	(*d*,*e*)		(2,0,0),(0,2,0),(0,0,2)	(0,0,0)
44 *Imm*2	(*a*,0)	(*b*,0,0)	(*c*)	(*d*,*e*)		(1,−1,0),(1,1,0),(0,0,2)	(0,0,0)
							
					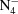 (**k** = 1/2,1/2,0)		
Derived from 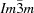							
63 *Cmcm*	(*a*,*a* 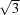 )	(0,*b*,0)	(0)	(0,0)	(0,0,0,0,*c*,0)	(1,0,0),(0,1,−1),(0,1,1)	(0,1/2,0)
63 *Cmcm*	(*a*,0)	(*b*,0,0)	(0)	(0,0)	(*c*,0,0,0,0,0)	(0,0,1),(1,−1,0),(1,1,0)	(0,1/2,1/2)
12 *C*2/*m*	(*a*,*b*)	(0,*c*,0)	(0)	(0,0)	(0,0,0,0,*d*,0)	(0,−1,1),(1,0,0),(0,1,1)	(1/2,1/2,0)
							
Derived from 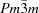							
51 *Pmma*	(*a*,*a* 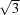 )	(0,*b*,0)	(*c*)	(0,0)	(0,0,0,0,*d*,0)	(0,1,1),(1,0,0),(0,1,−1)	(0,1/2,0)
51 *Pmma*	(*a*,0)	(*b*,0,0)	(*c*)	(0,0)	(*d*,0,0,0,0,0)	(1,1,0),(0,0,1),(1,−1,0)	(1/2,0,0)
10 *P*2/*m*	(*a*,*b*)	(0,*c*,0)	(*d*)	(0,0)	(0,0,0,0,*e*,*f*)	(0,1,1),(1,0,0),(0,1,−1)	(0,1/2,0)
							
Derived from 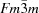							
59 *Pmmn*	(*a*,*a* 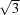 )	(0,*b*,0)	(*c*)	(0,*d*)	(0,0,0,0,*e*,0)	(2,0,0),(0,1,1),(0,−1,1)	(0,1/2,0)
59 *Pmmn*	(*a*,0)	(*b*,0,0)	(*c*)	(0,*d*)	(*e*,0,0,0,0,0)	(0,0,2),(1,1,0),(−1,1,0)	(1/2,0,0)
11 *P*2_1_/*m*	(*a*,*b*)	(0,*c*,0)	(*d*)	(0,*e*)	(0,0,0,0,*f*,*g*)	(0,−1,1),(2,0,0),(0,1,1)	(0,0,1/2)
							
Derived from 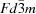							
62 *Pnma*	(*a*,*a* 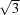 )	(0,*b*,0)	(0)	(*c*,−*c*)	(0,0,0,0,*d*,0)	(0,1,−1),(0,1,1),(2,0,0)	(3/4,3/4,3/4)
62 *Pnma*	(*a*,0)	(*b*,0,0)	(0)	(*c*,−*c*)	(*d*,0,0,0,0,0)	(1,−1,0),(1,1,0),(0,0,2)	(3/4,3/4,3/4)
14 *P*2_1_/*c*	(*a*,*b*)	(0,*c*,0)	(0)	(*d*,−*d*)	(0,0,0,0,*e*,0)	(0,1,1),(2,0,0),(0,1,−1)	(3/4,1/4,1/4)
							
Derived from 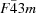							
31 *Pmn*2_1_	(*a*,*a* 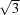 )	(0,*b*,0)	(*c*)	(*d*,*e*)	(0,0,0,0,*f*,0)	(0,1,1),(0,−1,1),(2,0,0)	(0,3/4,1/4)
31 *Pmn*2_1_	(*a*,0)	(*b*,0,0)	(*c*)	(*d*,*e*)	(*f*,0,0,0,0,0)	(1,1,0),(−1,1,0),(0,0,2)	(3/4,1/4,0)
4 *P*2_1_	(*a*,*b*)	(0,*c*,0)	(*d*)	(*e*,*f*)	(0,0,0,0,*g*,*h*)	(0,−1,1),(2,0,0),(0,1,1))	(0,0,1/2)
							
Derived from 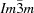							
					Σ_2_ (**k** = 1/3,1/3,0)		
42 *Fmm*2	(*a*,0)	(*b*,0,0)	(0)	(0,0)	(0,*c*,0,0,0,0,0,0,0,0,0,0)	(0,0,1),(3,3,0),(−1,1,0)	(0,0,0)
12 *C*2/*m*	(*a*,*b*)	(*c*,0,0)	(0)	(0,0)	(*d*,0,0,0,0,0,0,0,0,0,0,0)	(−1,1,0),(0,0,1),(1,2,0)	(0,0,0)
							
					Σ_2_ (**k** = 1/4,1/4,0)		
63 *Cmcm*	(*a*,0)	(*b*,0,0)	(0)	(0,0)	(*c*,0,0,0,0,0,0,0,0,0,0,0)	(0,0,1),(1,−1,0),(2,2,0)	(0,0,0)
12 *C*2/*m*	(*a*,*b*)	(*c*,0,0)	(0)	(0,0)	(*d*,0,0,0,0,0,0,0,0,0,0,0)	(−1,1,0),(0,0,1),(2,2,0)	(0,0,0)
64 *Cmca*	(*a*,0)	(*b*,0,0)	(0)	(0,0)	(*c*,−*c*,0,0,0,0,0,0,0,0,0,0)	(0,0,1),(1,−1,0),(2,2,0)	(0,1/2,1/2)
12 *C*2/*m*	(*a*,*b*)	(*c*,0,0)	(0)	(0,0)	(*d*,−*d*,0,0,0,0,0,0,0,0,0,0)	(−1,1,0),(0,0,1),(2,2,0)	(1/2,0,1/2)
							
					Σ_2_ (**k** = 1/5,1/5,0)		
42 *Fmm*2	(*a*,0)	(*b*,0,0)	(0)	(0,0)	(0,*c*,0,0,0,0,0,0,0,0,0,0)	(0,0,1),(5,5,0),(−1,1,0)	(0,0,0)
12 *C*2/*m*	(*a*,*b*)	(*c*,0,0)	(0)	(0,0)	(*d*,0,0,0,0,0,0,0,0,0,0,0)	(−1,1,0),(0,0,1),(2,3,0)	(0,0,0)
							
					Σ_2_ (**k** = 1/6,1/6,0)		
64 *Cmca*	(*a*,0)	(*b*,0,0)	(0)	(0,0)	(*c*,0,0,0,0,0,0,0,0,0,0,0)	(0,0,1),(1,−1,0),(3,3,0)	(0,0,0)
12 *C*2/*m*	(*a*,*b*)	(*c*,0,0)	(0)	(0,0)	(*d*,0,0,0,0,0,0,0,0,0,0,0)	(−1,1,0),(0,0,1),(3,3,0)	(0,0,0)
63 *Cmcm*	(*a*,0)	(*b*,0,0)	(0)	(0,0)	(*c*,−*c*/ 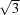 ,0,0,0,0,0,0,0,0,0,0)	(0,0,1),(1,−1,0),(3,3,0)	(0,1/2,1/2)
12 *C*2/*m*	(*a*,*b*)	(*c*,0,0)	(0)	(0,0,0)	(*d*,−*d*/ 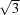 ,0,0,0,0,0,0,0,0,0,0)	(−1,1,0),(0,0,1),(3,3,0)	(1/2,0,1/2)
							
					Σ_2_ (**k** = 1/7,1/7,0)		
42 *Fmm*2	(*a*,0)	(*b*,0,0)	(0)	(0,0)	(0,*c*,0,0,0,0,0,0,0,0,0,0)	(0,0,1),(7,7,0),(−1,1,0)	(0,0,0)
12 *C*2/*m*	(*a*,*b*)	(*c*,0,0)	(0)	(0,0)	(*d*,0,0,0,0,0,0,0,0,0,0,0)	(−1,1,0),(0,0,1),(3,4,0)	(0,0,0)
							
					Σ_2_ (**k** = ξ,ξ,0) (incommensurate)		
69.1.17.2 *Fmmm*(0,0,γ)*s*00	(*a*,0)	(*b*,0,0)	(0)	(0,0)	(*c*,0,0,0,0,0,0,0,0,0,0,0)	(−1,1,0,0),(0,0,−1,0),(−1,−1,0,0),(0,0,0,1)	(0,0,0,0)
12.1.4.1 *B*2/*m*(α,β,0)00	(*a*,*b*)	(*c*,0,0)	(0)	(0,0)	(*d*,0,0,0,0,0,0,0,0,0,0,0)	(−1,1,0,0),(1,−2,0,0),(0,0,1,0),(0,0,0,1)	(0,0,0,0)
							
Derived from 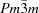							
					Σ_2_ (**k** = 1/3,1/3,0)		
38 *Amm*2	(*a*,0)	(*b*,0,0)	(*c*)	(0,0)	(0,*d*,0,0,0,0,0,0,0,0,0,0)	(0,0,1),(3,3,0),(−1,1,0)	(0,0,0)
10 *P*2/*m*	(*a*,*b*)	(*c*,0,0)	(*d*)	(0,0)	(*e*,0,0,0,0,0,0,0,0,0,0,0)	(−1,1,0),(0,0,1),(2,1,0)	(0,0,0)
							
					Σ_2_ (**k** = 1/4,1/4,0)		
51 *Pmma*	(*a*,0)	(*b*,0,0)	(*c*)	(0,0)	(*d*,0,0,0,0,0,0,0,0,0,0,0)	(2,2,0),(0,0,1),(1,−1,0)	(0,0,0)
10 *P*2/*m*	(*a*,*b*)	(*c*,0,0)	(*d*)	(0,0)	(*e*,0,0,0,0,0,0,0,0,0,0,0)	(−1,1,0),(0,0,1),(2,2,0)	(0,0,0)
55 *Pbam*	(*a*,0)	(*b*,0,0)	(*c*)	(0,0)	(*d*,−*d*,0,0,0,0,0,0,0,0,0,0)	(1,−1,0),(2,2,0),(0,0,1)	(1/2,0,0)
10 *P*2/*m*	(*a*,*b*)	(*c*,0,0)	(*d*)	(0,0)	(*e*,−*e*,0,0,0,0,0,0,0,0,0,0)	(−1,1,0),(0,0,1),(2,2,0)	(0,1/2,0)
							
					Σ_2_ (**k** = 1/5,1/5,0)		
38 *Amm*2	(*a*,0)	(*b*,0,0)	(*c*)	(0,0)	(0,*d*,0,0,0,0,0,0,0,0,0,0)	(0,0,1),(5,5,0),(−1,1,0)	(0,0,0)
10 *P*2/*m*	(*a*,*b*)	(*c*,0,0)	(*d*)	(0,0)	(*e*,0,0,0,0,0,0,0,0,0,0,0)	(−1,1,0),(0,0,1),(3,2,0)	(0,0,0)
							
					Σ_2_ (**k** = 1/6,1/6,0)		
55 *Pbam*	(*a*,0)	(*b*,0,0)	(*c*)	(0,0)	(*d*,0,0,0,0,0,0,0,0,0,0,0)	(1,−1,0),(3,3,0),(0,0,1)	(0,0,0)
10 *P*2/*m*	(*a*,*b*)	(*c*,0,0)	(*d*)	(0,0)	(*e*,0,0,0,0,0,0,0,0,0,0,0)	(−1,1,0),(0,0,1),(3,3,0)	(0,0,0)
51 *Pmma*	(*a*,0)	(*b*,0,0)	(*c*)	(0,0)	(*d*,−*d*/ 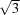 ,0,0,0,0,0,0,0,0,0,0)	(3,3,0),(0,0,1),(1,−1,0)	(1/2,0,0)
10 *P*2/*m*	(*a*,*b*)	(*c*,0,0)	(*d*)	(0,0)	(*e*,−*e*/ 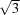 ,0,0,0,0,0,0,0,0,0,0)	(−1,1,0),(0,0,1),(3,3,0)	(0,1/2,0)
							
					Σ_2_ (**k** = 1/7,1/7,0)		
38 *Amm*2	(*a*,0)	(*b*,0,0)	(*c*)	(0,0)	(0,*d*,0,0,0,0,0,0,0,0,0,0)	(0,0,1),(7,7,0),(−1,1,0)	(0,0,0)
10 *P*2/*m*	(*a*,*b*)	(*c*,0,0)	(*d*)	(0,0)	(*e*,0,0,0,0,0,0,0,0,0,0,0)	(−1,1,0),(0,0,1),(4,3,0)	(0,0,0)
							
					Σ_2_ **k** = (ξ,ξ,0) (incommensurate)		
65.1.15.10 *Ammm*(0,0,γ)0*s*0	(*a*,0)	(*b*,0,0)	(*c*)	(0,0)	(*d*,0,0,0,0,0,0,0,0,0,0,0)	(0,0,1,0),(−1,1,0,0),(−1,−1,0,0),(0,0,0,1)	(0,0,0,0)
10.1.2.1 *P*2/*m*(α,β,0)00	(*a*,*b*)	(*c*,0,0)	(*d*)	(0,0)	(*e*,0,0,0,0,0,0,0,0,0,0,0)	(0,1,0,0),(−1,0,0,0),(0,0,1,0),(0,0,0,1)	(0,0,0,0)
							
Derived from 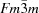							
					Σ_2_ (**k** = 1/3,1/3,0)		
44 *Imm*2	(*a*,0)	(*b*,0,0)	(*c*)	(0,*d*)	(0,*e*,0,0,0,0,0,0,0,0,0,0)	(0,0,2),(3,3,0),(−1,1,0)	(0,0,1/2)
12 *C*2/*m*	(*a*,*b*)	(*c*,0,0)	(*d*)	(0,*e*)	(*f*,0,0,0,0,0,0,0,0,0,0,0)	(2,4,0),(0,0,2),(1,−1,0)	(0,0,1/2)
							
					Σ_2_ (**k** = 1/4,1/4,0)		
51 *Pmma*	(*a*,0)	(*b*,0,0)	(*c*)	(0,*d*)	(0,*e*,0,0,0,0,0,0,0,0,0,0)	(2,2,0),(0,0,2),(1,−1,0)	(1/2,1/2,1/2)
10 *P*2/*m*	(*a*,*b*)	(*c*,0,0)	(*d*)	(0,*e*)	(0,*f*,0,0,0,0,0,0,0,0,0,0)	(−1,1,0),(0,0,2),(2,2,0)	(1/2,1/2,1/2)
62 *Pnma*	(*a*,0)	(*b*,0,0)	(*c*)	(0,*d*)	(*e*,−*e*,0,0,0,0,0,0,0,0,0,0)	(2,2,0),(0,0,2),(1,−1,0)	(0,1/2,0)
11 *P*2_1_/*m*	(*a*,*b*)	(*c*,0,0)	(*d*)	(0,*e*)	(*f*,−*f*,0,0,0,0,0,0,0,0,0,0)	(−1,1,0),(0,0,2),(2,2,0)	(0,1/2,0)
							
					Σ_2_ (**k** = 1/5,1/5,0)		
44 *Imm*2	(*a*,0)	(*b*,0,0)	(*c*)	(0,*d*)	(0,*e*,0,0,0,0,0,0,0,0,0,0)	(0,0,2),(5,5,0),(−1,1,0)	(0,0,1/2)
12 *C*2/*m*	(*a*,*b*)	(*c*,0,0)	(*d*)	(0,*e*)	(*f*,0,0,0,0,0,0,0,0,0,0,0)	(4,6,0),(0,0,2),(1,−1,0)	(0,0,1/2)
							
					Σ_2_ (**k** = 1/6,1/6,0)		
58 *Pnnm*	(*a*,0)	(*b*,0,0)	(*c*)	(0,*d*)	(*e*,−*e* 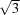 ,0,0,0,0,0,0,0,0,0,0)	(1,−1,0),(3,3,0),(0,0,2)	(1/2,1/2,1/2)
10 *P*2/*m*	(*a*,*b*)	(*c*,0,0)	(*d*)	(0,*e*)	(*f*,−*f* 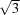 ,0,0,0,0,0,0,0,0,0,0)	(−1,1,0),(0,0,2),(3,3,0)	(1/2,1/2,1/2)
59 *Pmmn*	(*a*,0)	(*b*,0,0)	(*c*)	(0,*d*)	(*e*,−*e*/ 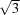 ,0,0,0,0,0,0,0,0,0,0)	(0,0,2),(3,3,0),(−1,1,0)	(0,1/2,0)
11 *P*2_1_/*m*	(*a*,*b*)	(*c*,0,0)	(*d*)	(0,*e*)	(*f*,−*f*/ 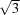 ,0,0,0,0,0,0,0,0,0,0)	(−1,1,0),(0,0,2),(3,3,0)	(0,1/2,0)
							
					Σ_2_ (**k** = 1/7,1/7,0)		
44 *Imm*2	(*a*,0)	(*b*,0,0)	(*c*)	(0,*d*)	(0,*e*,0,0,0,0,0,0,0,0,0,0)	(0,0,2),(7,7,0),(−1,1,0)	(0,0,1/2)
12 *C*2/*m*	(*a*,*b*)	(*c*,0,0)	(*d*)	(0,*e*)	(*f*,0,0,0,0,0,0,0,0,0,0,0)	(6,8,0),(0,0,2),(1,−1,0)	(0,0,1/2)
							
					Σ_2_ (**k** = ξ,ξ,0) (incommensurate)		
71.1.12.2 *Immm*(0,0,γ)*s*00	(*a*,0)	(*b*,0,0)	(*c*)	(*d*,0)	(*e*,0,0,0,0,0,0,0,0,0,0,0)	(1,−1,0,0),(0,0,2,0),(−1,−1,0,0),(0,0,0,1)	(0,0,0,0)
12.1.4.1 *B*2/*m*(α,β,0)00	(*a*,*b*)	(*c*,0,0)	(*d*)	(*e*,0)	(*f*,0,0,0,0,0,0,0,0,0,0,0	(0,2,0,0),(−1,−1,0,0),(0,0,2,0),(0,0,0,1)	(0,0,0,0)
							
Derived from 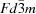							
					Σ_2_ (**k** = 1/3,1/3,0)		
46 *Ima*2	(*a*,0)	(*b*,0,0)	(0)	(*c*,−*c*)	(0,*d*,0,0,0,0,0,0,0,0,0,0)	(3,3,0),(0,0,2),(1,−1,0)	(−1,−1/2,−5/4)
15 *C*2/*c*	(*a*,*b*)	(*c*,0,0)	(0)	(*d*,−*d*)	(*e*,0,0,0,0,0,0,0,0,0,0,0)	(2,4,0),(0,0,2),(1,−1,0)	(1/4,−1/4,−3/4)
							
					Σ_2_ (**k** = 1/4,1/4,0)		
57 *Pbcm*	(*a*,0)	(*b*,0,0)	(0)	(*c*,−*c*)	(*d*,0,0,0,0,0,0,0,0,0,0,0)	(0,0,2),(1,−1,0),(2,2,0)	(1/4,−1/4,1/4)
13 *P*2/*c*	(*a*,*b*)	(*c*,0,0)	(0)	(*d*,−*d*)	(*e*,0,0,0,0,0,0,0,0,0,0,0)	(2,2,0),(0,0,2),(1,−1,0)	(1/4,−1/4,1/4)
60 *Pbcn*	(*a*,0)	(*b*,0,0)	(0)	(*c*,−*c*)	(*d*,*d*,0,0,0,0,0,0,0,0,0,0)	(0,0,2),(1,−1,0),(2,2,0)	(3/4,3/4,3/4)
14 *P*2_1_/*c*	(*a*,*b*)	(*c*,0,0)	(0)	(*d*,−*d*)	(*e*,*e*,0,0,0,0,0,0,0,0,0,0)	(2,2,0),(0,0,2),(1,−1,0)	(3/4,3/4,3/4)
							
					Σ_2_ (**k** = 1/5,1/5,0)		
46 *Ima*2	(*a*,0)	(*b*,0,0)	(0)	(*c*,−*c*)	(0,*d*,0,0,0,0,0,0,0,0,0,0)	(5,5,0),(0,0,2),(1,−1,0)	(−3/2,−1,−5/4)
15 *C*2/*c*	(*a*,*b*)	(*c*,0,0)	(0)	(*d*,−*d*)	(*e*,0,0,0,0,0,0,0,0,0,0,0)	(4,6,0),(0,0,2),(1,−1,0)	(1/4,−1/4,−3/4)
							
					Σ_2_ (**k** = 1/6,1/6,0)		
52 *Pnna*	(*a*,0)	(*b*,0,0)	(0)	(*c*,−*c*)	(*d*,0,0,0,0,0,0,0,0,0,0,0)	(1,−1,0),(3,3,0),(0,0,2)	(1/4,−1/4,1/4)
13 *P*2/*c*	(*a*,*b*)	(*c*,0,0)	(0)	(*d*,−*d*)	(*e*,0,0,0,0,0,0,0,0,0,0,0)	(3,3,0),(0,0,2),(1,−1,0)	(1/4,−1/4,1/4)
62 *Pnma*	(*a*,0)	(*b*,0,0)	(0)	(*c*,−*c*)	(0,*d*,0,0,0,0,0,0,0,0,0,0)	(1,−1,0),(3,3,0),(0,0,2)	(3/4,3/4,3/4)
14 *P*2_1_/c	(*a*,*b*)	(*c*,0,0)	(0)	(*d*,−*d*)	(0,*e*,0,0,0,0,0,0,0,0,0,0)	(3,3,0),(0,0,2),(1,−1,0)	(3/4,3/4,3/4)
							
					Σ_2_ (**k** = 1/7,1/7,0)		
46 *Ima*2	(*a*,0)	(*b*,0,0)	(0)	(*c*,−*c*)	(0,*d*,0,0,0,0,0,0,0,0,0,0)	(7,7,0),(0,0,2),(1,−1,0)	(−2,−3/2,−5/4)
15 *C*2/*c*	(*a*,*b*)	(*c*,0,0)	(0)	(*d*,−*d*)	(*e*,0,0,0,0,0,0,0,0,0,0,0)	(6,8,0),(0,0,2),(1,−1,0)	(1/4,−1/4,−3/4)
							
					Σ_2_ (**k** = ξ,ξ,0) (incommensurate)		
74.1.12.7 *Icmm*(0,0,γ)0*s*0	(*a*,0)	(*b*,0,0)	(0)	(*c*,*c*)	(*d*,0,0,0,0,0,0,0,0,0,0,0)	(0,0,2,0),(−1,1,0,0),(−1,−1,0,0),(0,0,0,1)	(−1/4,1/4,−5/4,0)
15.1.4.1 *B*2/*b*(α,β,0)00	(*a*,*b*)	(*c*,0,0)	(0)	(*d*,*d*)	(*e*,0,0,0,0,0,0,0,0,0,0,0)	(0,2,0,0),(1,−1,0,0),(0,0,−2,0),(0,0,0,1)	(1/4,−1/4,3/4,0)
							
Derived from 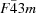							
					Σ_2_ (**k** = 1/3,1/3,0)		
8 *Cm*	(*a*,0)	(*b*,*c*,−*c*)	(*d*)	(*e*,*f*)	(0,*g*,0,0,0,0,0,0,0,0,0,0)	(1,−1,2),(3,3,0),(−1,1,0)	(0,0,0)
5 *C*2	(*a*,*b*)	(*c*,0,0)	(*d*)	(*e*,*f*)	(*g*,0,0,0,0,0,0,0,0,0,0,0)	(2,4,0),(0,0,2),(1,−1,0)	(0,0,0)
							
					Σ_2_ (**k** = 1/4,1/4,0)		
28 *Pma*2	(*a*,0)	(*b*,0,0)	(*c*)	(*d*,e)	(*f*,0,0,0,0,0,0,0,0,0,0,0)	(2,2,0),(−1,1,0),(0,0,2)	(0,0,0)
3 *P*2	(*a*,*b*)	(*c*,0,0)	(*d*)	(*e*,*f*)	(*g*,0,0,0,0,0,0,0,0,0,0,0)	(−1,1,0),(0,0,2),(2,2,0)	(0,0,0)
33 *Pna*2_1_	(*a*,0)	(*b*,0,0)	(*c*)	(*d*,e)	(*f*,−*f*,0,0,0,0,0,0,0,0,0,0)	(2,2,0),(−1,1,0),(0,0,2)	(0,1/2,0)
4 *P*2_1_	(*a*,*b*)	(*c*,0,0)	(*d*)	(*e*,*f*)	(*g*,−*g*,0,0,0,0,0,0,0,0,0,0)	(−1,1,0),(0,0,2),(2,2,0)	(0,1/2,0)
							
					Σ_2_ (**k** = 1/5,1/5,0)		
8 *Cm*	(*a*,0)	(*b*,*c*,−*c*)	(*d*)	(*e*,*f*)	(0,*g*,0,0,0,0,0,0,0,0,0,0)	(1,−1,2),(5,5,0),(−1,1,0)	(0,0,0)
5 *C*2	(*a*,*b*)	(*c*,0,0)	(*d*)	(*e*,*f*)	(*g*,0,0,0,0,0,0,0,0,0,0,0)	(4,6,0),(0,0,2),(1,−1,0)	(0,0,0)
							
					Σ_2_ (**k** = 1/6,1/6,0)		
34 *Pnn*2	(*a*,0)	(*b*,0,0)	(*c*)	(*d*,*e*)	(*f*,0,0,0,0,0,0,0,0,0,0,0)	(1,−1,0),(3,3,0),(0,0,2)	(0,0,0)
3 *P*2	(*a*,*b*)	(*c*,0,0)	(*d*)	(*e*,*f*)	(*g*,0,0,0,0,0,0,0,0,0,0,0)	(−1,1,0),(0,0,2),(3,3,0)	(0,0,0)
31 *Pmn*2_1_	(*a*,0)	(*b*,0,0)	(*c*)	(*d*,*e*)	(*f*,−*f* 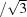 ,0,0,0,0,0,0,0,0,0,0)	(3,3,0),(−1,1,0),(0,0,2)	(3/4,5/4,0)
4 *P*2_1_	(*a*,*b*)	(*c*,0,0)	(*d*)	(*e*,*f*)	(*g*,−*g* 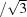 ,0,0,0,0,0,0,0,0,0,0)	(−1,1,0),(0,0,2),(3,3,0)	(0,1/2,0)
							
					Σ_2_ (**k** = 1/7,1/7,0)		
8 *Cm*	(*a*,0)	(*b*,*c*,−*c*)	(*d*)	(*e*,*f*)	(0,*g*,0,0,0,0,0,0,0,0,0,0)	(1,−1,2),(7,7,0),(−1,1,0)	(0,0,0)
5 *C*2	(*a*,*b*)	(*c*,0,0)	(*d*)	(*e*,*f*)	(*g*,0,0,0,0,0,0,0,0,0,0,0)	(6,8,0),(0,0,2),(1,−1,0)	(0,0,0)
							
					Σ_2_ (**k** = ξ,ξ,0) (incommensurate)		
44.1.12.5 *I*2*mm*(0,0,γ)0*s*0	(*a*,0)	(*b*,0,0)	(*c*)	(*d*,e)	(*f*,0,0,0,0,0,0,0,0,0,0,0)	(0,0,2,0),(−1,1,0,0),(−1,−1,0,0),(0,0,0,1)	(0,0,0,0)
5.1.4.1 *B*2(α,β,0)0	(*a*,*b*)	(*c*,0,0)	(*d*)	(*e*,*f*)	(*g*,0,0,0,0,0,0,0,0,0,0,0)	(0,2,0,0),(−1,−1,0,0),(0,0,2,0),(0,0,0,1)	(0,0,0,0)

**Table 3:** Symmetry relationships, order parameters and unit-cell configurations for selected subgroups of space group 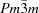 Labels in the last column are taken from the literature, including, in particular, from Otsuka *et al.* (1993 ▸).

	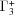	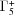	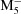	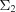	*k*-active	Basis vector	Origin	Approximate unit cell in relation to parent cubic cell	Other labels
221 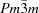								*a*_o_	B2
123 *P*4/*mmm*	(*a,*0)				(0,0,0)	(1,0,0),(0,1,0),(0,0,1)	(0,0,0)	*a*_o_, *a*_o_, *a*_o_	L1_0_
47 *Pmmm*	(*a*,*b*)				(0,0,0)	(1,0,0),(0,1,0),(0,0,1)	(0,0,0)	*a*_o_, *a*_o_, *a*_o_	
65 *Cmmm*	(*a*,0)	(*b*,0,0)			(0,0,0)	(1,1,0),(−1,1,0),(0,0,1)	(0,0,0)	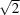 *a*_o_, 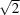 *a*_o_, *a*_o_	
									
51 *Pmma*	(*a*,− 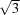 *a*)	(0,0,*b*)	(0,0,*c*,*c*,0,0)		(1/2,0,1/2)	(1,0,−1),(0,1,0),(1,0,1)	(1/2,0,0)	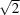 *a*_o_, *a*_o_, 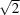 *a*_o_	B19, 2H or 2O
10 *P*2/*m*	(*a*,*b*)	(0,0,*c*)	(0,0,*d*,*e*,0,0)		(1/2,0,1/2)	(1,0,−1),(0,1,0),(1,0,1)	(1/2,0,0)	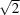 *a*_o_, *a*_o_, 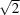 *a*_o_	3R or 2M
11 *P*2_1_/*m*	(*a*,− 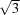 *a*)	(*b*,*b*,*c*)	(0,0,*d*,*d*,0,0)		(0,0,0), (1/2,0,1/2)	(0,1,0),(−1,0,1),(1,0,1)	(0,0,1/2)	*a*_o_, 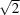 *a*_o_, 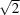 *a*_o_	B19′
									
147 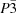	(0,0)	(*a*,−*a*,*a*)		(*b*,0,0,0,*b*,0,0,0,0,0,*b*,0)	(1/3,1/3,0),(1/3,0,1/3), (0,1/3,−1/3)	(1,−1,2),(1,2,−1),(−1,1,1)	(0,0,0)	Rhombohedral cell: 3 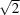 *a*_o_, 3 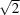 *a*_o_, 3 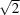 *a*_o_	R-phase
38 *Amm*2	(*a*,0)	(*b*,0,0)		(0,*c*,0,0,0,0,0,0,0,0,0,0)	(1/3,1/3,0)	(0,0,1),(3,3,0),(−1,1,0)	(0,0,0)	*a*_o_, 3 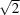 *a*_o_, 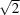 *a*_o_	
10 *P*2/*m*	(*a*,*b*)	(*c*,0,0)		(*d*,0,0,0,0,0,0,0,0,0,0,0)	(1/3,1/3,0)	(−1,1,0),(0,0,1),(2,1,0)	(0,0,0)	Monoclinic cell: 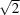 *a*_o_, *a*_o_, 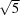 *a*_o_, β ≃ [90 + tan^−1^(1/3)]° = 108°, orthorhombic pseudo-cell: 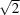 *a*_o_, *a*_o_, 3 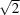 *a*_o_, (β ≃ 90°)	9R or 6M
									
51 *Pmma*	(*a*,0)	(*b*,0,0)		(*c*,0,0,0,0,0,0,0,0,0,0,0)	(1/4,1/4,0)	(2,2,0),(0,0,1),(1,−1,0)	(0,0,0)	2 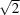 *a*_o_, *a*_o_, 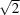 *a*_o_	
10 *P*2/*m*	(*a*,*b*)	(*c*,0,0)		(*d*,0,0,0,0,0,0,0,0,0,0,0)	(0,0,0) (1/4,1/4,0)	(−1,1,0),(0,0,1),(2,2,0)	(0,0,0)	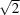 *a*_o_, *a*_o_, 2 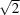 *a*_o_	
55 *Pbam*	(*a*,0)	(*b*,0,0)	(*c*,*c*,0,0,0,0)	(*d*,−*d*,0,0,0,0,0,0,0,0,0,0)	(1/4,1/4,0)	(1,−1,0),(2,2,0),(0,0,1)	(1/2,0,0)	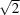 *a*_o_, 2 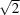 *a*_o_, *a*_o_	
10 *P*2/*m*	(*a*,*b*)	(*c*,0,0)	(*d*,*e*,0,0,0,0)	(*f*,−*f*,0,0,0,0,0,0,0,0,0,0)	(0,0,0) (1/4,1/4,0)	(−1,1,0),(0,0,1),(2,2,0)	(0,1/2,0)	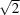 *a*_o_, *a*_o_, 2 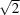 *a*_o_	
									
38 *Amm*2	(*a*,0)	(*b*,0,0)		(0,*c*,0,0,0,0,0,0,0,0,0,0)	(1/5,1/5,0)	(0,0,1),(5,5,0),(−1,1,0)	(0,0,0)	*a*_o_, 5 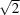 *a*_o_, 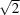 *a*_o_	
10 *P*2/*m*	(*a*,*b*)	(*c*,0,0)		(*d*,0,0,0,0,0,0,0,0,0,0,0)	(1/5,1/5,0)	(−1,1,0),(0,0,1),(3,2,0)	(0,0,0)	Monoclinic cell: 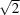 *a*_o_, *a*_o_, 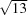 *a*_o_, β ≃ [90 + tan^−1^(1/5)]° = 101°, orthorhombic pseudo-cell: 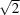 *a*_o_, *a*_o_, 5 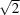 *a*_o_, (β ≃ 90°)	5M or 10M
									
55 *Pbam*	(*a*,0)	(*b*,0,0)		(*c*,0,0,0,0,0,0,0,0,0,0,0)	(1/6,1/6,0)	(1,−1,0),(3,3,0),(0,0,1)	(0,0,0)	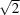 *a*_o_, 3 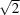 *a*_o_, *a*_o_	
10 *P*2/*m*	(*a*,*b*)	(*c*,0,0)		(*d*,0,0,0,0,0,0,0,0,0,0,0)	(0,0,0) (1/6,1/6,0)	(−1,1,0),(0,0,1),(3,3,0)	(0,0,0)	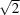 *a*_o_, *a*_o_, 3 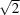 *a*_o_	
51 *Pmma*	(*a*,0)	(*b*,0,0)	(*c*,−*c*,0,0,0,0)	(*d*,−*d*/ 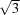 ,0,0,0,0,0,0,0,0,0,0)	(1/6,1/6,0)	(3,3,0),(0,0,1),(1,−1,0)	(1/2,0,0)	3*a* 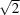 _o_, 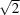 *a*_o_, *a*_o_	
10 *P*2/*m*	(*a*,*b*)	(*c*,0,0)	(*d*,*e*,0,0,0,0)	(*f*,−*f*/ 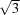 ,0,0,0,0,0,0,0,0,0,0)	(0,0,0) (1/6,1/6,0)	(−1,1,0),(0,0,1),(3,3,0)	(0,1/2,0)	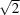 *a*_o_, *a*_o_, 3 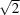 *a*_o_	
									
38 *Amm*2	(*a*,0)	(*b*,0,0)		(0,*c*,0,0,0,0,0,0,0,0,0,0)	(1/7,1/7,0)	(0,0,1),(7,7,0),(−1,1,0)	(0,0,0)	*a*_o_, 7 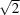 *a*_o_, 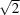 *a*_o_	
10 *P*2/*m*	(*a*,*b*)	(*c*,0,0)		(*d*,0,0,0,0,0,0,0,0,0,0,0)	(1/7,1/7,0)	(−1,1,0),(0,0,1),(4,3,0)	(0,0,0)	Monoclinic cell: 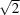 *a*_o_, *a*_o_, 5*a*_o_, β ≃ [90 + tan^−1^(1/7)]° = 98°, orthorhombic pseudo-cell: 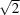 *a*_o_, *a*_o_, 7 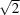 *a*_o_, (β ≃ 90°)	7R, 7M or 14M
									
Incommensurate									
65.1.15.10 *Ammm*(0,0,γ)0*s*0	(*a*,0)	(*b*,0,0)		(*c*,0,0,0,0,0,0,0,0,0,0,0)	(ξ,ξ,0)	(0,0,1,0),(−1,1,0,0), (−1,−1,0,0),(0,0,0,1)	(0,0,0,0)	*a*_o_, 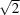 *a*_o_, 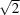 *a*_o_/ 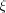	IC
10.1.2.1 *P*2/*m*(α,β,0)00	(*a*,*b*)	(*c*,0,0)		(*d*,0,0,0,0,0,0,0,0,0,0,0)	(0,0,0), (ξ,ξ,0)	(0,1,0,0),(−1,0,0,0), (0,0,1,0),(0,0,0,1)	(0,0,0,0)		

**Table 4:** Symmetry relationships, order parameters and unit cell configurations for selected subgroups of space group 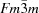 Note that components of the *k*-active vector are a factor of two larger here than for the same structures in Table 2 ▸, due to the fact that the parent 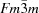 structure has a unit cell with dimensions twice those of the 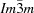 parent cell. For the same reason, the lattice vectors listed to describe the origin and basis are halved relative to those shown in Table 2 ▸. Finally, we note that the origin of space group 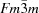 is at (1/2,1/2,1/2) with respect to the 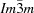 cell.

	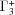	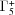	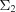	*k*-active	Basis vector	Origin	Approximate unit cell in relation to parent cubic cell
225 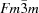							*a*_oF_
139 *I*4/*mmm*	(*a*,0)			(0,0,0)	(1/2,1/2,0),(−1/2,1/2,0),(0,0,1)	(0,0,0)	*a*_oF_/ 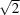 , *a*_oF_/ 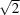 , *a*_oF_
69 *Fmmm*	(*a*,*b*)			(0,0,0)	(1,0,0),(0,1,0),(0,0,1)	(0,0,0)	*a*_oF_
71 *Immm*	(*a*,0)	(*b*,0,0)		(0,0,0)	(1/2,1/2,0),(−1/2,1/2,0),(0,0,1)	(0,0,0)	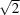 *a*_oF_, 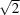 *a*_oF_, *a*_oF_
							
51 *Pmma*	(*a*,0)	(*b*,0,0)	(*c*,0,0,0,0,0,0,0,0,0,0,0)	(1/2,1/2,0)	(1,1,0),(0,0,1),(1/2,−1/2,0)	(0,0,0)	*a*_oF_/ 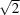 , *a*_oF_/ 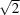 , *a*_oF_
10 *P*2/*m*	(*a*,*b*)	(*c*,0,0)	(*d*,0,0,0,0,0,0,0,0,0,0,0)	(0,0,0) (1/2,1/2,0)	(−1/2,1/2,0),(0,0,1),(1,1,0)	(0,0,0)	*a*_oF_/ 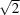 , *a*_oF_, 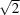 *a*_oF_
62 *Pnma*	(*a*,0)	(*b*,0,0)	(*c*,−*c*,0,0,0,0,0,0,0,0,0,0)	(1/2,1/2,0)	(1,1,0),(0,0,1),(1/2,−1/2,0)	(1/4,0,1/4)	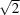 *a*_oF_, *a*_oF_, *a*_oF_/ 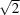
11 *P*2_1_/*m*	(*a*,*b*)	(*c*,0,0)	(*d*,−*d*,0,0,0,0,0,0,0,0,0,0)	(0,0,0) (1/2,1/2,0)	(−1/2,1/2,0),(0,0,1),(1,1,0)	(1/4,0,1/4)	*a*_oF_/ 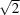 , *a*_oF_, 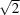 *a*_oF_
							
58 *Pnnm*	(*a*,0)	(*b*,0,0)	(*c*,0,0,0,0,0,0,0,0,0,0,0)	(1/3,1/3,0)	(1/2,−1/2,0),(3/2,3/2,0),(0,0,1)	(0,0,0)	*a*_oF_/ 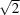 , 3*a*_oF_/ 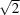 , *a*_oF_
10 *P*2/*m*	(*a*,*b*)	(*c*,0,0)	(*d*,0,0,0,0,0,0,0,0,0,0,0)	(0,0,0) (1/3,1/3,0)	(−1/2,1/2,0),(0,0,1),(3/2,3/2,0)	(0,0,0)	*a*_oF_/ 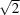 , *a*_oF_, 3*a*_oF_/ 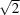
59 *Pmmn*	(*a*,0)	(*b*,0,0)	(*c*,−*c*/ 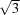 ,0,0,0,0,0,0,0,0,0,0)	(1/3,1/3,0)	(0,0,1),(3/2,3/2,0),(−1/2,1/2,0)	(1/4,0,1/4)	*a*_oF_, 3*a*_oF_/ 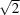 , *a*_oF_/ 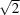
11 *P*2_1_/*m*	(*a*,*b*)	(*c*,0,0)	(*d*,−*d*/ 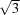 ,0,0,0,0,0,0,0,0,0,0)	(0,0,0) (1/3,1/3,0)	(−1/2,1/2,0),(0,0,1),(3/2,3/2,0)	(1/4,0,1/4)	*a*_oF_/ 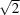 , *a*_oF_, 3*a*_oF_/ 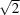
							
51 *Pmma*	(*a*,0)	(*b*,0,0)	(*c*,0,0,0,0,0,0,0,0,0,0,0)	(1/4,1/4,0)	(2,2,0),(0,0,1),(1/2,−1/2,0)	(0,0,0)	2 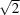 *a*_oF_, *a*_oF_, *a*_oF_/ 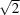
10 *P*2/*m*	(*a*,*b*)	(*c*,0,0)	(*d*,0,0,0,0,0,0,0,0,0,0,0)	(0,0,0) (1/4,1/4,0)	(−1/2,1/2,0),(0,0,1),(2,2,0)	(0,0,0)	*a*_oF_/ 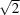 , *a*_oF_, 2 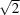 *a*_oF_
62 *Pnma*	(*a*,0)	(*b*,0,0)	(*c*,−0.414*c*,0,0,0,0,0,0,0,0,0,0)	(1/4,1/4,0)	(2,2,0),(0,0,1),(1/2,−1/2,0)	(1/4,0,1/4)	2 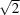 *a*_oF_, *a*_oF_, *a*_oF_/ 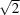
11 *P*2_1_/*m*	(*a*,*b*)	(*c*,0,0)	(*d*,−0.414*d*,0,0,0,0,0,0,0,0,0,0)	(0,0,0) (1/4,1/4,0)	(−1/2,1/2,0),(0,0,1),(2,2,0)	(1/4,0,1/4)	*a*_oF_/ 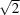 , *a*_oF_, 2 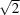 *a*_oF_
							
58 *Pnnm*	(*a*,0)	(*b*,0,0)	(*c*,0,0,0,0,0,0,0,0,0,0,0)	(1/5,1/5,0)	(1/2,−1/2,0),(5/2,5/2,0),(0,0,1)	(0,0,0)	*a*_oF_/ 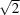 , 5*a*_oF_/ 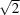 , *a*_oF_
10 *P*2/*m*	(*a*,*b*)	(*c*,0,0)	(*d*,0,0,0,0,0,0,0,0,0,0,0)	(0,0,0) (1/5,1/5,0)	(−1/2,1/2,0),(0,0,1),(5/2,5/2,0)	(0,0,0)	*a*_oF_/ 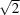 , *a*_oF_, 5*a*_oF_/ 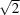
59 *Pmmn*	(*a*,0)	(*b*,0,0)	(0.951*c*, −0.309*c*,0,0,0,0,0,0,0,0,0,0)	(1/5,1/5,0)	(0,0,1),(5/2,5/2,0),(−1/2,1/2,0)	(1/4,0,1/4)†	*a*_oF_, 5*a*_oF_/ 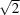 , *a*_oF_/ 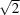
11 *P*2_1_/*m*	(*a*,*b*)	(*c*,0,0)	(0.951*d*,−0.309*d*,0,0,0,0,0,0,0,0,0)	(0,0,0) (1/5,1/5,0)	(−1/2,1/2,0),(0,0,1),(5/2,5/2,0)	(1/4,0,1/4)†	*a*_oF_/ 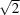 , *a*_oF_, 5*a*_oF_/ 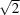
							
51 *Pmma*	(*a*,0)	(*b*,0,0)	(*c*,0,0,0,0,0,0,0,0,0,0,0)	(1/6,1/6,0)	(3,3,0),(0,0,1),(1/2,−1/2,0)	(0,0,0)	3 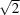 *a*_oF_, *a*_oF_, *a*_oF_/ 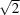
10 *P*2/*m*	(*a*,*b*)	(*c*,0,0)	(*d*,0,0,0,0,0,0,0,0,0,0,0)	(0,0,0) (1/6,1/6,0)	(−1/2,1/2,0),(0,0,1),(3,3,0)	(0,0,0)	*a*_oF_/ 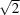 , *a*_oF_, 3 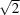 *a*_oF_
62 *Pnma*	(*a*,0)	(*b*,0,0)	(1.366*c*,−0.366*c*,0,0,0,0,0,0,0,0,0,0)	(1/6,1/6,0)	(3,3,0),(0,0,1),(1/2,−1/2,0)	(1/4,0,1/4)†	3 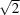 *a*_oF_, *a*_oF_, *a*_oF_/ 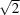
11 *P*2_1_/*m*	(*a*,*b*)	(*c*,0,0)	(1.366*d*,−0.366*d*,0,0,0,0,0,0,0,0,0,0)	(0,0,0) (1/6,1/6,0)	(−1/2,1/2,0),(0,0,1),(3,3,0)	(1/4,0,1/4)†	*a*_oF_/ 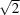 , *a*_oF_, 3 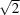 *a*_oF_
							
58 *Pnnm*	(*a*,0)	(*b*,0,0)	(*c*,0,0,0,0,0,0,0,0,0,0,0)	(1/7,1/7,0)	(1/2,−1/2,0),(7/2,7/2,0),(0,0,1)	(0,0,0)	*a*_oF_/ 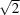 , 7*a*_oF_/ 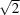 , *a*_oF_
10 *P*2/*m*	(*a*,*b*)	(*c*,0,0)	(*d*,0,0,0,0,0,0,0,0,0,0,0)	(0,0,0) (1/7,1/7,0)	(−1/2,1/2,0),(0,0,1),(7/2,7/2,0)	(0,0,0)	*a*_oF_/ 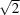 , *a*_oF_, 7*a*_oF_/ 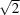
59 *Pmmn*	(*a*,0)	(*b*,0,0)	(0.975*c*,−0.223*c*,0,0,0,0,0,0,0,0,0,0)	(1/7,1/7,0)	(0,0,1),(7/2,7/2,0),(−1/2,1/2,0)	(1/4,0,1/4)†	*a*_oF_, 7*a*_oF_/ 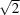 , *a*_oF_/ 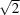
11 *P*2_1_/*m*	(*a*,*b*)	(*c*,0,0)	(0.975*d*,−0.223*d*,0,0,0,0,0,0,0,0,0,0)	(0,0,0) (1/7,1/7,0)	(−1/2,1/2,0),(0,0,1),(7/2,7/2,0)	(1/4,0,1/4)†	*a*_oF_/ 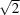 , *a*_oF_, 7*a*_oF_/ 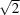
							
Incommensurate							
71.1.12.2 *Immm*(0,0,γ)s00	(*a*,0)	(*b*,0,0)	(*c*,0,0,0,0,0,0,0,0,0,0,0)	(ξ,ξ,0)	(1/2,−1/2,0,0),(0,0,1,0), (1/2,1/2,0,0),(0,0,0,1)	(0,0,0,0)	*a*_oF_/ 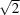 , *a*_oF_, *a*_oF_/ξ 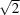
12.1.4.1 *B*2/*m*(α,β,0)00	(*a*,*b*)	(*c*,0,0)	(*d*,0,0,0,0,0,0,0,0,0,0,0)	(0,0,0) (ξ,ξ,0)	(0,1,0,0),(1/2,−3/2,0,0),(0,0,−1,0),(0,0,0,1)},	(0,0,0,0)	

† Domains other than the default domain provided by *ISOTROPY* have been selected in order to have a consistent origin of (1/4,0,1/4).

## Footnotes

1 Repeat distance is defined by the sequence of atomic displacements and may or may not correspond to a crystallographic repeat.
